# Reinforcement Learning Using a Continuous Time Actor-Critic Framework with Spiking Neurons

**DOI:** 10.1371/journal.pcbi.1003024

**Published:** 2013-04-11

**Authors:** Nicolas Frémaux, Henning Sprekeler, Wulfram Gerstner

**Affiliations:** 1 School of Computer and Communication Sciences and School of Life Sciences, Brain Mind Institute, École Polytechnique Fédérale de Lausanne, 1015 Lausanne EPFL, Switzerland; 2 Theoretical Neuroscience Lab, Institute for Theoretical Biology, Humboldt-Universität zu Berlin, Berlin, Germany; Université Paris Descartes, Centre National de la Recherche Scientifique, France

## Abstract

Animals repeat rewarded behaviors, but the physiological basis of reward-based learning has only been partially elucidated. On one hand, experimental evidence shows that the neuromodulator dopamine carries information about rewards and affects synaptic plasticity. On the other hand, the theory of reinforcement learning provides a framework for reward-based learning. Recent models of reward-modulated spike-timing-dependent plasticity have made first steps towards bridging the gap between the two approaches, but faced two problems. First, reinforcement learning is typically formulated in a discrete framework, ill-adapted to the description of natural situations. Second, biologically plausible models of reward-modulated spike-timing-dependent plasticity require precise calculation of the reward prediction error, yet it remains to be shown how this can be computed by neurons. Here we propose a solution to these problems by extending the continuous temporal difference (TD) learning of Doya (2000) to the case of spiking neurons in an actor-critic network operating in continuous time, and with continuous state and action representations. In our model, the critic learns to predict expected future rewards in real time. Its activity, together with actual rewards, conditions the delivery of a neuromodulatory TD signal to itself and to the actor, which is responsible for action choice. In simulations, we show that such an architecture can solve a Morris water-maze-like navigation task, in a number of trials consistent with reported animal performance. We also use our model to solve the acrobot and the cartpole problems, two complex motor control tasks. Our model provides a plausible way of computing reward prediction error in the brain. Moreover, the analytically derived learning rule is consistent with experimental evidence for dopamine-modulated spike-timing-dependent plasticity.

## Author Summary

As every dog owner knows, animals repeat behaviors that earn them rewards. But what is the brain machinery that underlies this reward-based learning? Experimental research points to plasticity of the synaptic connections between neurons, with an important role played by the neuromodulator dopamine, but the exact way synaptic activity and neuromodulation interact during learning is not precisely understood. Here we propose a model explaining how reward signals might interplay with synaptic plasticity, and use the model to solve a simulated maze navigation task. Our model extends an idea from the theory of reinforcement learning: one group of neurons form an “actor,” responsible for choosing the direction of motion of the animal. Another group of neurons, the “critic,” whose role is to predict the rewards the actor will gain, uses the mismatch between actual and expected reward to teach the synapses feeding both groups. Our learning agent learns to reliably navigate its maze to find the reward. Remarkably, the synaptic learning rule that we derive from theoretical considerations is similar to previous rules based on experimental evidence.

## Introduction

Many instances of animal behavior learning such as path finding in foraging, or – a more artificial example – navigating the Morris water-maze, can be interpreted as exploration and trial-and-error learning. In both examples, the behavior eventually learned by the animal is the one that led to high reward. These can be appetite rewards (i.e., food) or more indirect rewards, such as the relief of finding the platform in the water-maze.

Important progress has been made in understanding how learning of such behaviors takes place in the mammalian brain. On one hand, the framework of reinforcement learning [1] provides a theory and algorithms for learning with sparse rewarding events. A particularly attractive formulation of reinforcement learning is temporal difference (TD) learning [2]. In the standard setting, this theory assumes that an agent moves between states in its environment by choosing appropriate actions in discrete time steps. Rewards are given in certain conjunctions of states and actions, and the agent's aim is to choose its actions so as to maximize the amount of reward it receives. Several algorithms have been developed to solve this standard formulation of the problem, and some of these have been used with spiking neural systems. These include REINFORCE [3], [4] and partially observable Markov decision processes [5], [6], in case the agent has incomplete knowledge of its state.

On the other hand, experiments show that dopamine, a neurotransmitter associated with pleasure, is released in the brain when reward, or a reward-predicting event, occurs [7]. Dopamine has been shown to modulate the induction of plasticity in timing non-specific protocols [8]–[11]. Dopamine has also recently been shown to modulate spike-timing-dependent plasticity (STDP), although the exact spike-timing and dopamine requirements for induction of long-term potentiation (LTP) and long-term depression (LTD) are still unclear [12]–[14].

A crucial problem in linking biological neural networks and reinforcement learning is that typical formulations of reinforcement learning rely on discrete descriptions of states, actions and time, while spiking neurons evolve naturally in continuous time and biologically plausible “time-steps” are difficult to envision. Earlier studies suggested that an external reset [15] or theta oscillations [16] might be involved, but no evidence exists to support this and it is not clear why evolution would favor slower decision steps over a continuous decision mechanism. Indeed biological decision making is often modeled by an integrative process in continuous time [17], where the actual decision is triggered when the integrated value reaches a threshold.

In this study, we propose a way to narrow the conceptual gap between reinforcement learning models and the family of spike-timing-dependent synaptic learning rules by using continuous representations of state, actions and time, and by deriving biologically plausible synaptic learning rules. More precisely, we use a variation of the Actor-Critic architecture [1], [18] for TD learning. Starting from the continuous TD formulation by Doya [19], we derive reward-modulated STDP learning rules which enable a network of model spiking neurons to efficiently solve navigation and motor control tasks, with continuous state, action and time representations. This can be seen as an extension of earlier works [20], [21] to continuous actions, continuous time and spiking neurons. We show that such a system has a performance on par with that of real animals and that it offers new insight into synaptic plasticity under the influence of neuromodulators such as dopamine.

## Results

**Figure 1:**
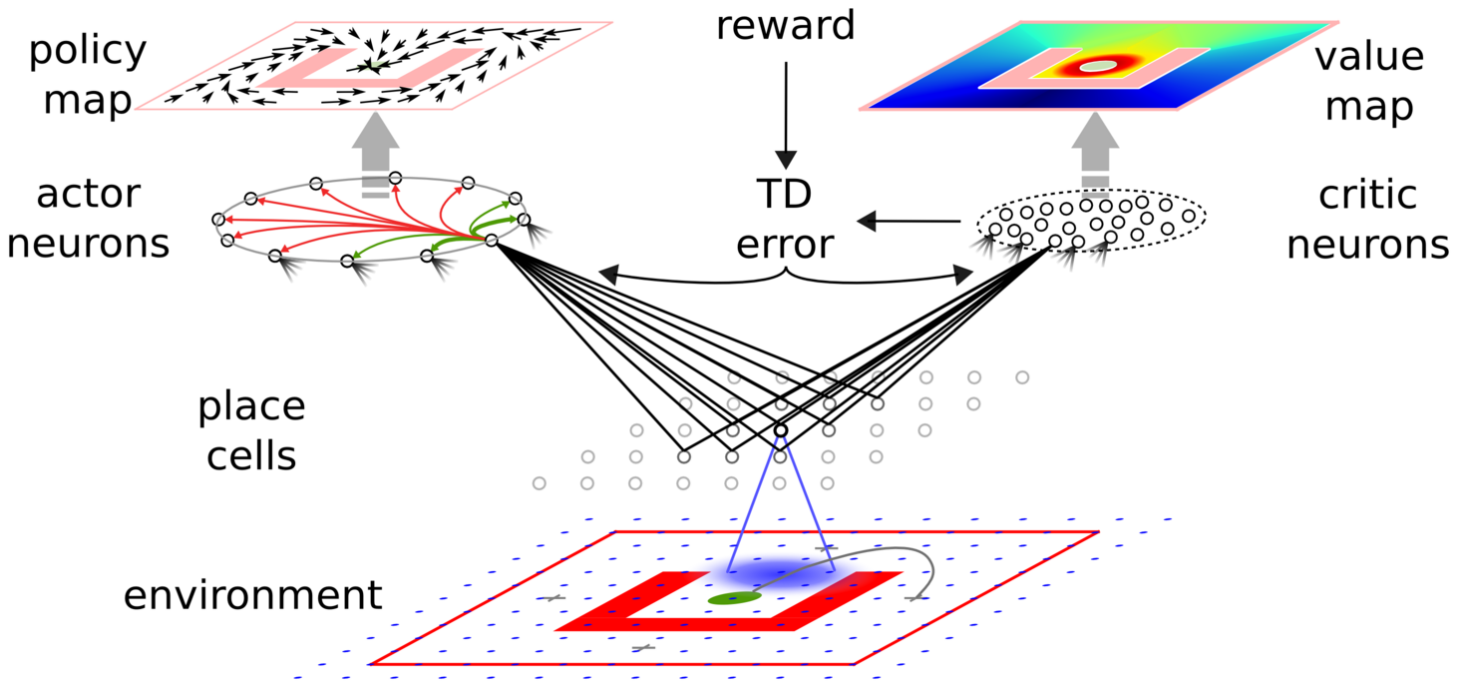
Navigation task and actor-critic network. From bottom to top: the simulated agent evolves in a maze environment, until it finds the reward area (green disk), avoiding obstacles (red). Place cells maintain a representation of the position of the agent through their tuning curves. Blue shadow: example tuning curve of one place cell (black); blue dots: tuning curves centers of other place cells. Right: a pool of critic neurons encode the expected future reward (value map, top right) at the agent's current position. The change in the predicted value is compared to the actual reward, leading to the temporal difference (TD) error. The TD error signal is broadcast to the synapses as part of the learning rule. Left: a ring of actor neurons with global inhibition and local excitation code for the direction taken by the agent. Their choices depending on the agent's position embody a policy map (top left).

How do animals learn to find their way through a maze? What kind of neural circuits underlie such learning and computation and what synaptic plasticity rules do they rely on? We address these questions by studying how a simulated animal (or *agent*) could solve a navigation task, akin to the Morris water-maze. Our agent has to navigate through a maze, looking for a (hidden) platform that triggers reward delivery and the end of the trial. We assume that our agent can rely on place cells [22] for a representation of its current position in the maze (Figure 1).

Temporal difference learning methods provide a theory explaining how an agent should interact with its environment to maximize the rewards it receives. TD learning is built on the formalism of Markov decision processes. In what follows, we reformulate the framework of Markov decision process in continuous time, state and action, before we turn to the actor-critic neural network and the learning rule we used to solve the maze task.

Let us consider a learning agent navigating through the maze. We can describe its position at time 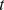 as 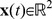, corresponding to a continuous version of the state in the standard reinforcement learning framework. The temporal evolution of the state is governed by the agent's action 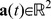, according to
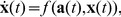
(1)where 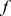 describes the dynamics of the environment. Throughout this paper we use the dot notation to designate the derivative of a term with respect to time.

We model place cells as simple spiking processes (inhomogeneous Poisson, see Models) that fire only when the agent approaches their respective center. The centers are arranged on a grid, uniformly covering the surface of the maze.

Reward is dispensed to the agent in the form of a reward rate 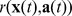. A localized reward 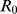 at a single position 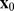 would correspond to the limit 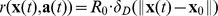, where 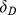 denotes the Dirac 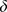-function. However, since any realistic reward (e.g., a piece of chocolate or the hidden platform in the water-maze) has a finite extent, we prefer to work with a temporally extended reward. In our model, rewards are attributed based on spatially precise events, but their delivery is temporally extended (see Models). The agent is rewarded for reaching the goal platform and punished (negative reward) for running into walls.

The agent follows a policy 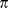 which determines the probability that an action 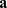 is taken in the state 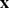


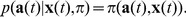
(2)The general aim of the agent is to find the policy 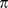 that ensures the highest reward return in the long run.

Several algorithms have been proposed to solve the discrete version of the reinforcement problem problem described above, such as Q-Learning [23] or Sarsa [24]. Both of these use a representation of the future rewards in form of 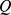-values for each state-action pair. The 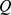-values are then used both to evaluate the current policy (*evaluation* problem) and to choose the next action (*control* problem). As we show in Models, 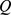-values lead to difficulties when one wishes to move to a continuous representation while preserving biological plausibility. Instead, here we use an approach dubbed “Actor-Critic” [1], [8], [21], where the agent is separated in two parts: the control problem is solved by an *actor* and the evaluation problem is solved by a *critic* (Figure 1).

The rest of the Results section is structured as follows. First we have a look at the TD formalism in continuous time. Next, we show how spiking neurons can implement a critic, to represent and learn the expected future rewards. Third, we discuss a spiking neuron actor, and how it can represent and learn a policy. Finally, simulation results show that our network successfully learns the simulated task.

### Continuous TD

The goal of a reinforcement learning agent is to maximize its future rewards. Following Doya [10], we define the continuous-time value function 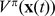 as
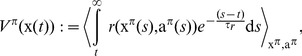
(3)where the brackets represent the expectation over all future trajectories 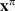 and future action choices 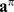, dependent on the policy 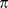. The parameter 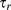 represents the reward discount time constant, analogous to the discount factor of discrete reinforcement learning. Its effect is to make rewards in the near future more attractive than distant ones. Typical values of 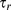 for a task such as the water-maze task would be on the order of a few seconds. Eq. 3 represents the total quantity of discounted reward that an agent in position 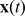 at time 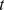 and following policy 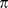 can expect. The policy should be chosen such that 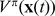 is maximized for all locations 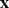. Taking the derivative of Eq. 3 with respect to time yields the self-consistency equation [19]

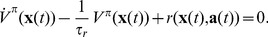
(4)


Calculating 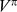 requires knowledge of the reward function 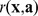 and of the environment dynamics 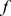 (Eq 1). These are, however, unknown to the agent. Typically, the best an agent can do is to maintain a parametric estimator 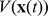 of the “true” value function 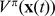. This estimator being imperfect, it is not guaranteed to satisfy Eq. 4. Instead, the temporal difference error 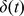 is defined as the mismatch in the self-consistency,
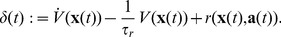
(5)This is analog to the discrete TD error [1], [19]

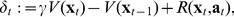
(6)where the reward discount factor 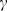 plays a role similar to the reward discount time constant 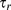. More precisely, for short steps 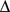, 
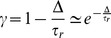
 [19].

An estimator 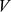 can be said to be a good approximation to 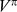 if the TD error 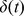 is close to zero for all 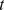. This suggests a simple way to learn a value function estimator: by a gradient descent on the squared TD error in the following way
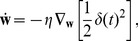
(7)where 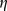 is a learning rate parameter and 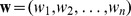 is the set of parameters (synaptic weights) that control the estimator 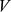 of the value function. This approach, dubbed residual gradient [19], [25], [26], yields a learning rule that is formally correct, but in our case suffers from a noise bias, as shown in Models.

Instead, we use a different learning rule, suggested for the discrete case by Sutton and Barto [1]. Translated in a continuous framework, the aim of their optimization approach is that the value function approximation 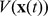 should match the true value function 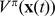. This is equivalent to minimizing an objective function
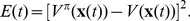
(8)A gradient descent learning rule on 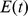 yields
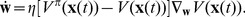
(9)Of course, because 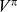 is unknown, this is not a particularly useful learning rule. On the other hand, using Eq. 4, this becomes
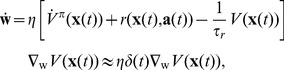
(10)where we merged 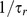 into the learning rate 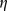 without loss of generality. In the last step, we replaced the real value function derivative with its estimate, i.e., 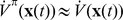, and then used the definition of 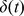 from Eq. 5.

The substitution of 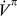 by 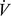 in Eq. 10 is an approximation, and there is in general no guarantee that the two values are similar. However the form of the resulting learning rule suggests it goes in the direction of reducing the TD error 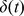. For example, if 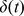 is positive at time 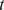, updating the parameters 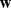 in the direction suggested by Eq. 10, will increase the value of 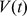, and thus decrease 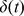.

In [19], a heuristic shortcut was used to go directly from the residual gradient (Eq. 7) to Eq. 10. As noted by Doya [19], the form of the learning rule in Eq. 10 is a continuous version of the discrete 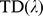 [1], [27] with function approximation (here with 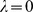). This has been shown to converge with probability 1 [28], [29], even in the case of infinite (but countable) state space. This must be the case also for arbitrarily small time steps (such as the finite steps usually used in computer simulations of a continuous system [19]), and thus it seems reasonable to expect that the continuous version also converges under reasonable assumptions, even though to date no proof exists.

An important problem in reinforcement learning is the concept of temporal credit assignment, i.e., how to propagate information about rewards back in time. In the framework of TD learning, this means propagating the TD error at time 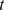 so that the value function at earlier times is updated in consequence. The learning rule Eq. 10 does not by itself offer a solution to this problem, because the expression of 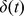 explicitly refers only to 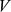 and 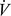 at time 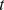. Therefore 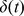 does not convey information about other times 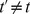 and minimizing 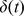 does not *a priori* affect values 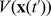 and 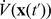. This is in contrast to the discrete version of the TD error (Eq. 6), where the expression of 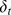 explicitly links to 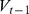 and thus the TD error is back-propagated during subsequent learning trials.

If, however, one assumes that the value function 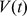 is continuous and continuously differentiable, changing the values of 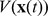 and 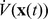 implies changing the values of these functions in a finite vicinity of 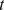. This is in particular the case if one uses a parametric form for 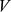, in the form of a weighted mixture of smooth kernels (as we do here, see next section). Therefore, the conjunction of a function approximation of the value function in the form of a linear combination of smooth kernels ensures that the TD error 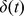 is propagated in time in the continuous case, allowing the temporal credit assignment problem to be solved.

### Spiking Neuron Critic

We now take the above derivation a step further by assuming that the value function estimation is performed by a spiking neuron with firing rate 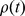. A natural way of doing this is
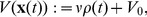
(11)where 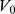 is the value corresponding to no spiking activity and 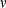 is a scaling factor with units of [reward units]×s. A choice of 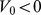 enables negative values 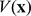, despite the fact that the rate 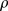 is always positive. We call this neuron a *critic neuron*, because its role is to maintain an estimate of the value function 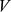.

Several aspects should be discussed at this point. Firstly, since the value function in Eq. 11 must depend on the state 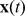 of the agent, we must assume that the neuron receives some meaningful synaptic input about the state of the agent. In the following we make the assumption that this input is feed-forward from the place cells to the (spiking) critic neuron.

Secondly, while the value function is in theory a function only of the state at time 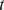, a spiking neuron implementation (such as the simplified model we use here, see Models) will reflect the recent past, in a manner determined by the shape of the excitatory postsynaptic potentials (EPSP) it receives. This is a limitation shared by all neural circuits processing sensory input with finite synaptic delays. In the rest of this study, we assume that the evolution of the state of the agent is slow compared to the width of an EPSP. In that limit, the firing rate of a critic neuron at time 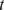 actually reflects the position of the agent at that time.

Thirdly, the firing rate 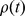 of a single spike-firing neuron is itself a vague concept and multiple definitions are possible. Let's start from its spike train 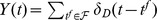 (where 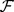 is the set of the neuron's spike times and 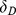 is the Dirac delta, not to be confused with the TD signal). The expectation 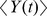 is a statistical average of the neuron's firing over many repetitions. It is the theoretically favored definition of the firing rate, but in practice it is not available in single trials in a biologically plausible setting. Instead, a common workaround is to use a temporal average, for example by filtering the spike train with a kernel 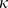


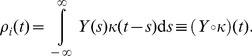
(12)Essentially, this amounts to a trade-off between temporal accuracy and smoothness of the rate function, of which extreme cases are respectively the spike train 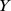 (extreme temporal accuracy) and a simple spike count over a long time window with smooth borders (no temporal information, extreme smoothness). In choosing a kernel 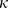, it should hold that 
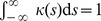
, so that each spike is counted once, and one often wishes the kernel to be causal (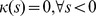), so that the current firing rate is fully determined by *past* spike times and independent of future spikes.

Another common approximation for the firing rate of a neuron consists in replacing the statistical average by a population average, over many neurons encoding the same value. Provided they are statistically independent of each other (for example if the neurons are not directly connected), averaging their responses over a single trial is equivalent to averaging the responses of a single neuron over the same number of trials.

Here we combine temporal and population averaging, redefining the value function as an average firing rate of 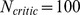 neurons
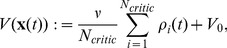
(13)where the instantaneous firing rate of neuron 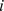 is defined by Eq. 12, using its spike train 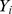 and a kernel 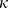 defined by
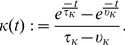
(14)This kernel rises with a time constant 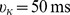 and decays to 0 with time constant 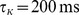. One advantage of the definition of Eq. 12 is that the derivative of the firing rate of neuron 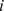 with respect to time is simply
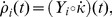
(15)so that computing the derivative of the firing rate is simply a matter of filtering the spike train with the derivative 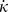 of the 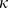 kernel. This way, the TD error 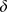 of Eq. 5 can be expressed as
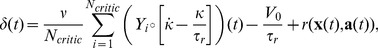
(16)where, again, 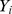 denotes the spike train of neuron 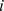 in the pool of critic neurons.

Suppose that feed-forward weights 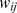 lead from a state-representation neuron 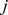 to neuron 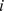 in the population of critic neurons. Can the critic neurons learn to approximate the value function by changing the synaptic weights? An answer to this question is obtained by combining Eq. 10 with Eqs 13 and 16, leading to a weights update
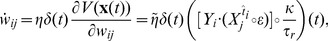
(17)where 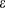 is the time course of an EPSP and 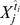 is the spike train of the presynaptic neuron 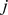, restricted to the spikes posterior to the last spike time 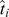 of postsynaptic neuron 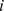. For simplicity, we merged all constants into a new learning rate 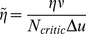. A more formal derivation can be found in Models.

**Figure 2:**
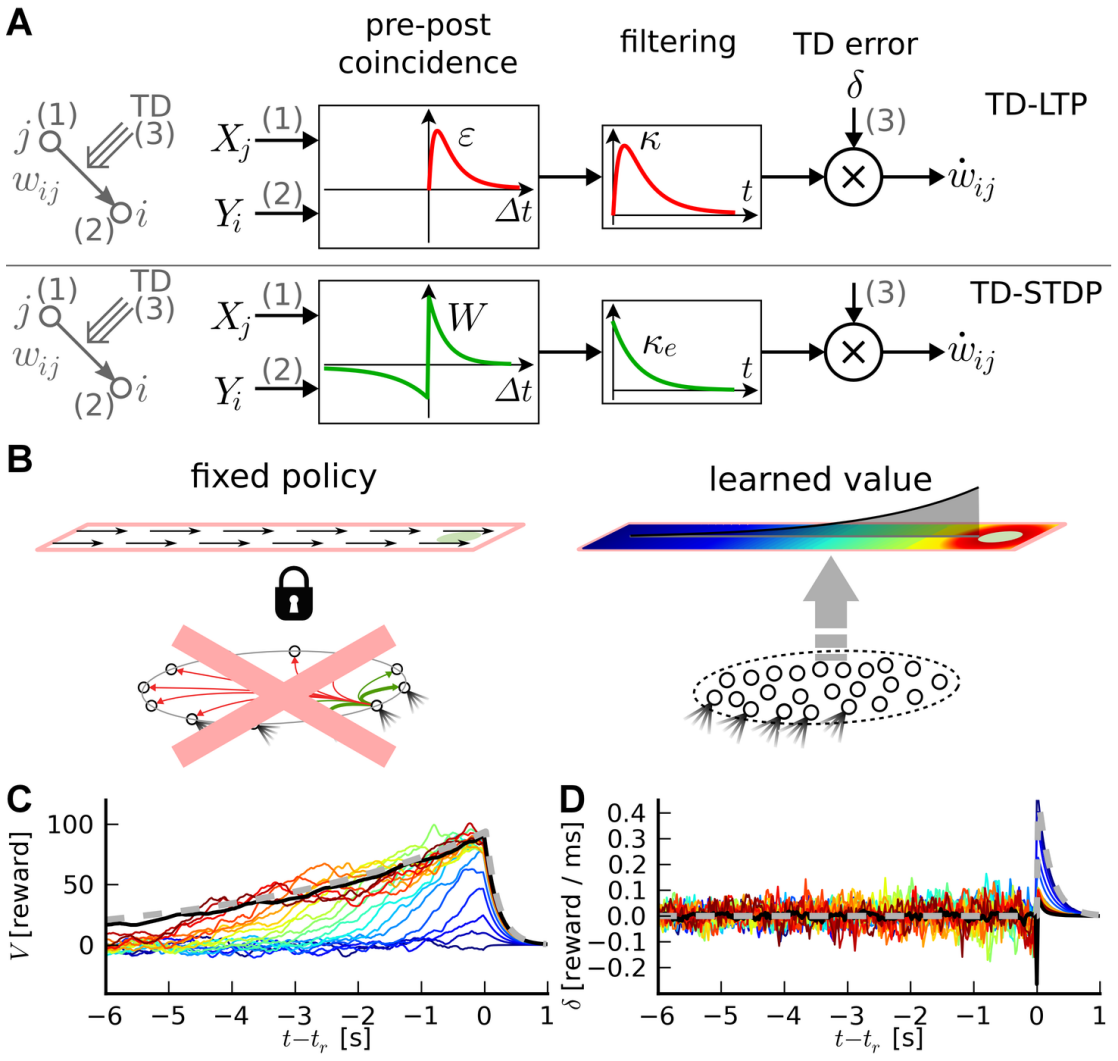
Critic learning in a linear track task. A: Learning rule with three factors. Top: TD-LTP is the learning rule given in Eq. 17. It works by passing the presynaptic spike train 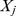 (factor 1) and the postsynaptic spike train 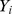 (factor 2) through a coincidence window 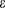. Spikes are counted as coincident if the postsynaptic spike occurs within after a few ms of a presynaptic spike. The result of the pre-post coincidence measure is filtered through a 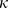 kernel, and then multiplied by the TD error 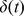 (factor 3) to yield the learning rule which controls the change 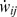 of the synaptic weight. Bottom: TD-STDP is a TD-modulated variant of R-STDP. The main difference with TD-LTP is the presence of a post-before-pre component in the coincidence window. B: Linear track task. The linear track experiment is a simplified version of the standard maze task. The actor's choice is forced to the correct direction with constant velocity (left), while the critic learns to represent value (right). C: Value function learning by the critic. Each colored trace shows the value function represented by the critic neurons activity against time in the 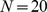 first simulation trials (from dark blue in trial 1 to dark red in trial 20), with 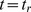 corresponding to the time of the reward delivery. The black line shows an average over trials 30 to 50, after learning converged. The gray dashed line shows the theoretical value function. D: TD signal 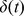 corresponding to the simulation in C. The gray dashed line shows the reward time course 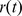.

Let us now have a closer look at the shape of the learning rule suggested by Eq. 17. The effective learning rate is given by a parameter 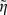. The rest of the learning rule consists of a product of two terms. The first one is the TD error term 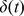, which is the same for all synapses 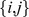, and can thus be considered as a global factor, possibly transmitted by one or more neuromodulators (Figure 1). This neuromodulator broadcasts information about inconsistency between the reward 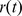 and the value function encoded by the population of critic neurons to all neurons in the network. The second term is synapse-specific and reflects the coincidence of EPSPs caused by presynaptic spikes of neuron 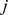 with the postsynaptic spikes of neuron 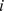. The postsynaptic term 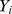 is a consequence of the exponential non-linearity used in the neuron model (see Models). This coincidence, “Hebbian” term is in turn filtered through the 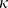 kernel which corresponds to the effect of a postsynaptic spike on 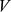. It reflects the responsibility of the synapse in the recent value function. Together these two terms form a three-factor rule, where the pre- and postsynaptic activities combine with the global signal 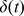 to modify synaptic strengths (Figure 2A, top). Because it has, roughly, the form of “TD error signal
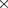
Hebbian LTP”, we call this learning rule TD-LTP.

We would like to point out the similarity of the TD-LTP learning rule to a reward-modulated spike-timing-dependent plasticity rule we call R-STDP [6], [16], [30]–[32]. In R-STDP, the effects of classic STDP [33]–[36] are stored into an exponentially decaying, medium term (time constant 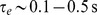), synapse-specific memory, called an *eligibility trace*. This trace is only imprinted into the actual synaptic weights when a global, neuromodulatory success signal 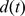 is sent to the synapses. In R-STDP, the neuromodulatory signal 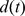 is the reward minus a baseline, i.e., 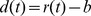. It was shown [32] that for R-STDP to maximize reward, the baseline must precisely match the mean (or expected) reward. In this sense, 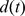 is a reward prediction error signal; a system to compute this signal is needed. Since the TD error is also a reward prediction error signal, it seems natural to use 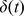 instead of 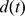. This turns the reward-modulated learning rule R-STDP into a TD error-modulated TD-STDP rule (Figure 2A, bottom). In this form, TD-STDP is very similar to TD-LTP. The major difference between the two is the influence of post-before-pre spike pairings on the learning rule: while these are ignored in TD-LTP, they cause a negative contribution to the coincidence detection in TD-STDP.

The filtering kernel 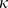, which was introduced to filter the spike trains into differentiable firing rates serves a role similar to the eligibility trace in R-STDP, and also in the discrete TD(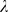) [1]. As noted in the previous section, this is the consequence of the combination of a smooth parametric function approximation of the value function (each critic spike contributes a shape 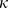 to 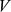) and the form of the learning rule from Eq. 10. The filtering kernel 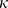 is crucial to back-propagation of the TD error, and thus to the solving of the temporal credit assignment problem.

### Linear Track Simulation

Having shown how spiking neurons can represent and learn the value function, we next test these results through simulations. However, in the actor-critic framework, the actor and the critic learn in collaboration, making it hard to disentangle the effects of learning in either of the two. To isolate learning by the critic and disregard potential problems of the actor, we temporarily sidestep this difficulty by using a forced action setup. We transform the water-maze into a linear track, and “clamp” the action choice to a value which leads the agent straight to the reward. In other words, the actor neurons are not simulated, see Figure 2B, and the agent simply “runs” to the goal. Upon reaching it at time 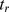, a reward is delivered and the trial ends.

Figure 2C shows the value function over 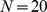 color-coded trials (from blue to red) as learned by a critic using the learning rule we described above. On the first run (dark blue trace), the critic neurons are naive about the reward and therefore represent a (noisy version of a) zero value function. Upon reaching the goal, the TD error (Figure 2D) matches the reward time course, 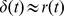. According to the learning rule in Eq. 17, this causes strengthening of those synapses that underwent pre-post activity recently before the reward (with “recent” defined by the 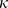 kernel). This is visible already at the second trial, when the value 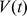 just before reward becomes positive.

In the next trials, this effect repeats, until the TD error vanishes. Suppose that, in a specific trials, reward starts at the time 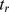 when the agent has reached the goal. According to the definition of the TD error, for all times 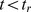 the 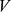-value is self consistent only if 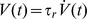 — or equivalently 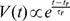. The gray dashed line in Figure 2C shows the time course of the theoretical value function; over many repetitions the colored traces, representing the value function in the different trials, move closer and closer to the theoretical value. The black line in Figure 2C represents the average value function over 20 late trials, after learning has converged: it nicely matches the theoretical value.

An interesting point that appears in Figure 2C is the clearly visible back-propagation of information about the reward expressed in the shape of the value function. In the first trials, the value function 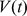 rises only for a short time just prior to the reward time. This causes, in the following trial, a TD error at earlier times. As trials proceed, synaptic weights corresponding to even earlier times increase. After 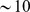 trials in Figure 2C, the value function roughly matches the theoretical value just prior to 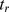, but not earlier. In subsequent trials, the point of mismatch is pushed back in time.

This back-propagation phenomenon is a signature of TD learning algorithms. Two things should be noted here. Firstly, the speed with which the back-propagation occurs is governed by the shape of the 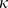 kernel in the Hebbian part of the learning rule. It plays a role equivalent to the eligibility trace in reinforcement learning: it “flags” a synapse after it underwent pre-before-post activity with a decaying trace, a trace that is only consolidated into a weight change when a global confirmation signal 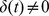 arrives. This “eligibility trace” role of 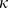 is distinct from its original role in the 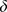 term, where it is used to smooth the spiking activity of the critic neurons (Eq. 12). As such, one might be tempted to change the decay time constant of the 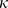 term in the learning rule so as to control back-propagation speed, while keeping the “other” 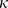 of the 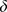 signal fixed. In separate simulations (not shown), we found that such an ad-hoc approach did not lead to a gain in learning performance.

Secondly, we know by construction that this back-propagation of the reward information is driven by the TD error signal 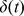. However, visual inspection of Figure 2D, which shows the 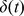 traces corresponding to the experiment in Figure 2C, does not reveal any clear back-propagation of the TD error. For 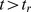, a large peak mirroring the reward signal 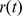 (gray dashed line) is visible in the early traces (blue lines) and recedes quickly as the value function correctly learns to expect the reward. For 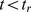, the 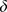 is dominated by fast noise, masking any back-propagation of the error signal, even though the fact that the value function is learned properly shows it is indeed present and effective. One might speculate that if a biological system was using such a TD error learning system with spiking neuron, and if an experimenter was to record a handful of critic neurons he would be at great pain to measure any significant TD error back-propagation. This is a possible explanation for the fact that no back-propagation signal has been observed in experiments.

We have already discussed the structural similarity of a TD-modulated version of the R-STDP rule [6], [30], [31] with TD-LTP. Simulations of the linear track experiment with the TD-STDP rule show that it behaves similarly to our learning rule (data not shown), i.e., the difference between the two rules (the post-before-pre part of the coincidence detection window, see Figure 2A) does not appear to play a crucial role in this case.

### Spiking Neuron Actor

We have seen above that spiking neurons in the “critic” population can learn to represent the expected rewards. We next ask how a spiking neuron agent chooses its actions so as to maximize the reward.

In the classical description of reinforcement learning, actions, like states and time, are discrete. While discrete actions can occur, for example when a laboratory animal has to choose which lever to press, most motor actions, such as hand reaching or locomotion in space, are more naturally described by continuous variables. Even though an animal only has a finite number of neurons, neural coding schemes such as *population vector coding* [37] allow a discrete number of neurons to code for a continuum of actions.

**Figure 3:**
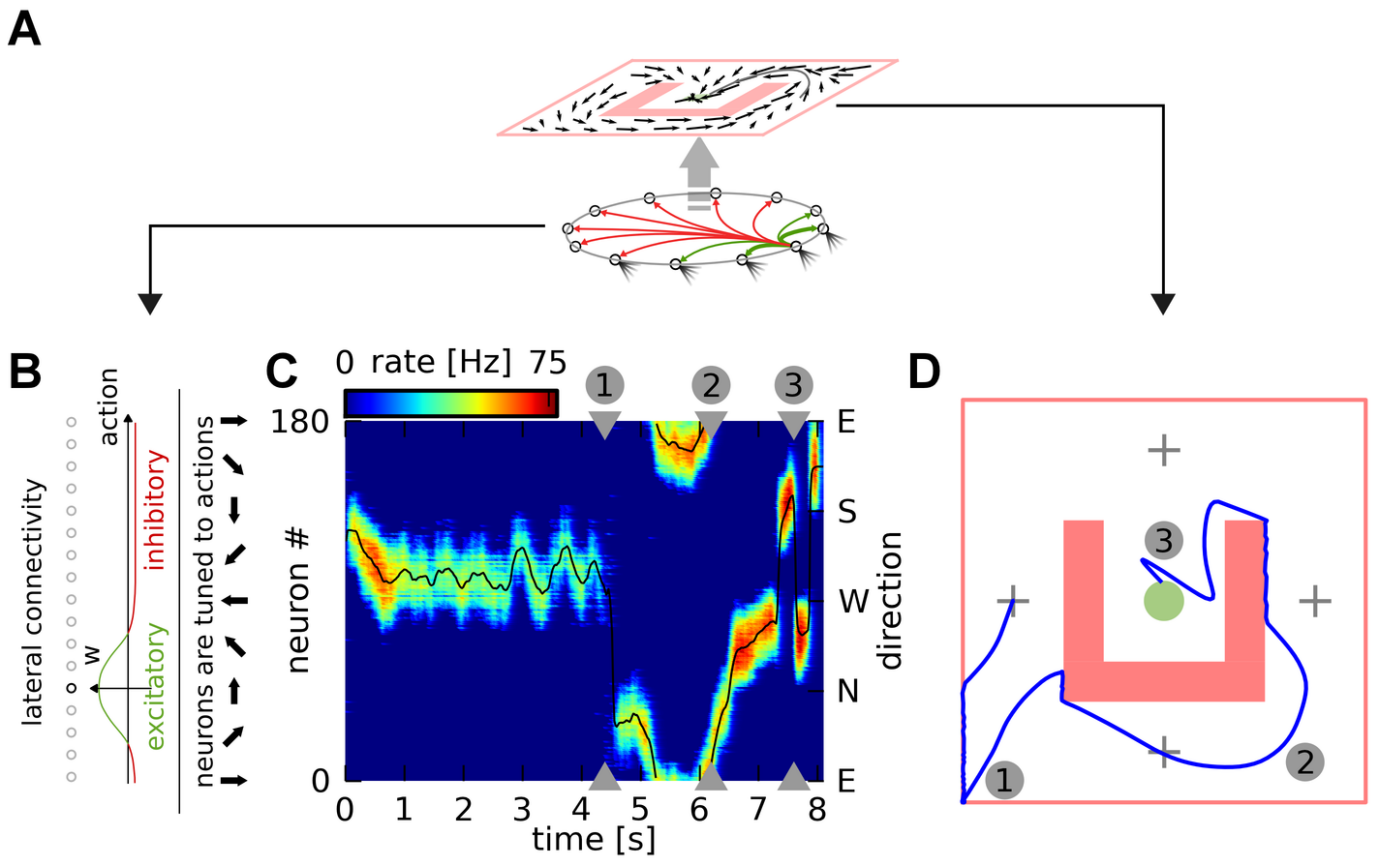
Actor neurons. A: A ring of actor neurons with lateral connectivity (bottom, green: excitatory, red: inhibitory) embodies the agent's policy (top). B: Lateral connectivity. Each neuron codes for a distinct motion direction. Neurons form excitatory synapses to similarly tuned neurons and inhibitory synapses to other neurons. C: Activity of actor neurons during an example trial. The activity of the neurons (vertical axis) is shown as a color map against time (horizontal axis). The lateral connectivity ensures that there is a single bump of activity at every moment in time. The black line shows the direction of motion (right axis; arrows in panel B) chosen as a result of the neural activity. D: Maze trajectory corresponding to the trial shown in C. The numbered position markers match the times marked in C.

We follow the population coding approach and define the actor as a group of 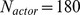 spiking neurons (Figure 3A), each coding for a different direction of motion. Like the critic neurons, these actor neurons receive connections from place cells, representing the current position of the agent. The spike trains generated by these neurons are filtered to produce a smooth firing rate, which is then multiplied by each neuron's preferred direction (see Models for all calculation details). We finally sum these vectors to obtain the actual agent action at that particular time. To ensure a clear choice of actions, we use a 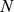-winner-take-all lateral connectivity scheme: each neuron excites the neurons with similar tuning and inhibits all other neurons (Figure 3B). We manually adjusted the connection strength so that there was always a single “bump” of neurons active. An example of the activity in the pool of actor neurons and the corresponding action readout over a (successful) trial is given in Figure 3C. The corresponding maze trajectory is shown in Figure 3D.

In reinforcement learning, a successful agent has to balance exploration of unvisited states and actions in the search for new rewards, and exploitation of previously successful strategies. In our network, the exploration/exploitation balance is the result of the bump dynamics. To see this, let us consider a naive agent, characterized by uniform connections from the place cells to the actor neurons. For this agent, the bump first forms at random and then drifts without preference in the action space. This corresponds to random action choices, or full exploration. After the agent has been rewarded for reaching the goal, synaptic weights linking particular place cells to a particular action will be strengthened. This will increase the probability that the bump forms for that action the next time over. Thus the action choice will become more deterministic, and the agent will exploit the knowledge it has acquired over previous trials.

Here, we propose to use the same learning rule for the actor neurons' synapses as for those of the critic neurons. The reason is the following. Let us look at the case where 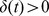: the critic is signaling that the recent sequence of actions taken by the agent has caused an unexpected reward. This means that the association between the action neurons that have recently been active and the state neurons whose input they have received should be strengthened so that the same action is more likely to be taken again in the next occurrence of that state. In the contrary case of a negative reinforcement signal, the connectivity to recently active action neurons should be weakened so that recently taken action are less likely to be taken again, leaving the way to, hopefully, better alternatives. This is similar to the way in which the synapses from the state input to the critic neurons should be strengthened or weakened, depending on their pre- and postsynaptic activities. This suggests that the action neurons should use the same synaptic learning rule as the one in Eq. 17, with 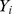 now denoting the activity of the action neurons, but the 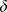 signal still driven by the critic activity. This is biologically plausible and consistent with our assumption that 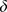 is communicated by a neuromodulator, which broadcasts information over a large fraction of the brain.

There are two critical effects of our 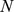-winner-take-all lateral connectivity scheme. Firstly, it ensures that only neurons coding for similar actions can be active at the same time. Because of the Hebbian part of the learning rule, this means that only those which are directly responsible for the action choice are subject to reinforcement, positive or negative. Secondly, by forcing the activity of the action neurons to take the shape of a group of similarly tuned neurons, it effectively causes generalization across actions: neurons coding for actions similar to the one chosen will also be active, and thus will also be given credit for the outcome of the action [16]. This is similar to the way the actor learns in non-neural actor-critic algorithms [18], [19], where only actions actually taken are credited by the learning rule. Thus, although an infinite number of actions are possible at each position, the agent does not have to explore every single one of them (an infinitely long task!) to learn the right strategy.

The fact that both the actor and the critic use the same learning rule is in contrast with the original formulation of the actor-critic network of Barto et al. [18], where the critic learning rule is of the form “TD error×presynaptic activity”. As discussed above, the “TD error×Hebbian LTP” form of the critic learning rule Eq. 17 used here is a result of the exponential non-linearity used in the neuron model. Using the same learning rule for the critic and the actor has the interesting property that a single biological plasticity mechanism has to be postulated to explain learning in both structures.

### Water-Maze Simulation

**Figure 4:**
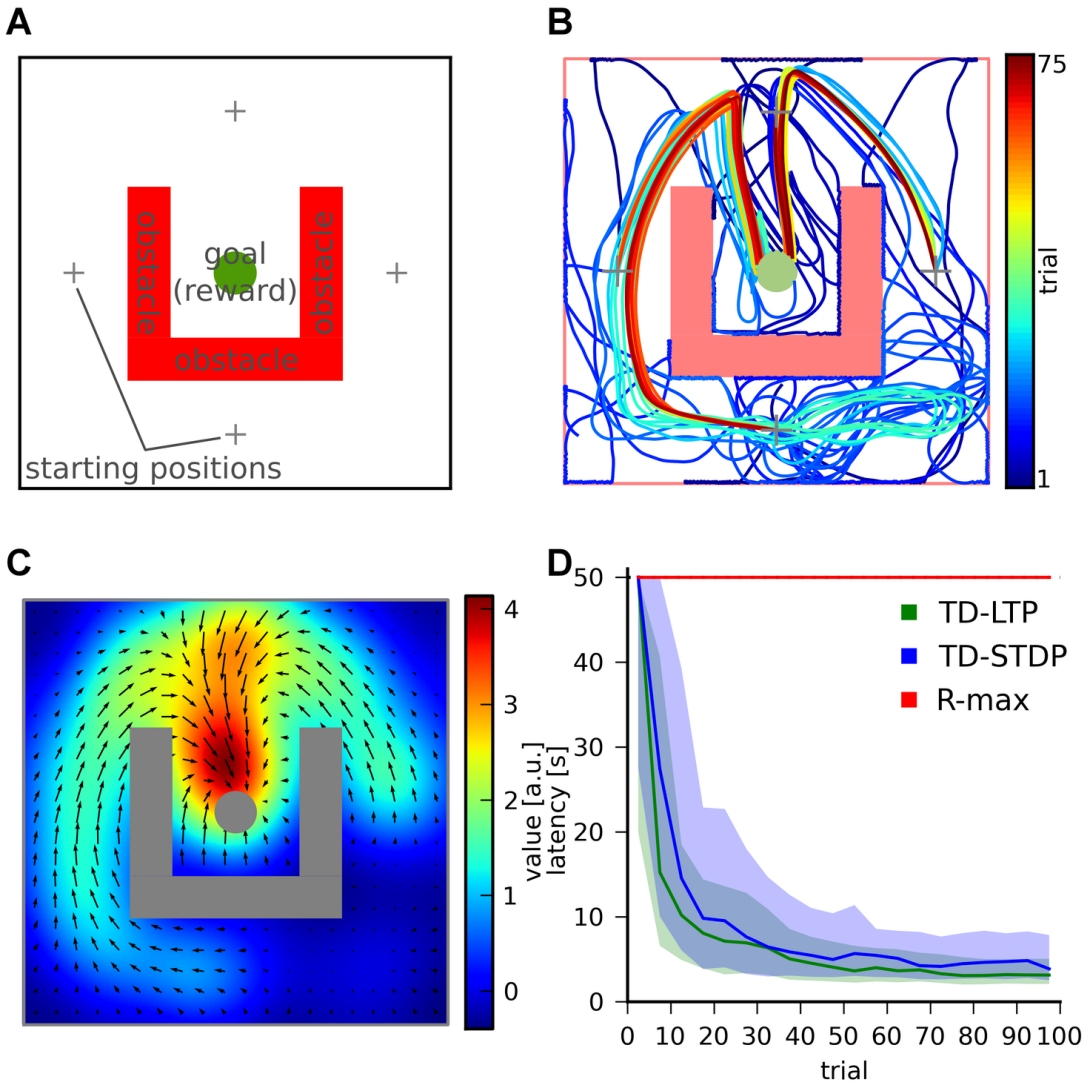
Maze navigation learning task. A: The maze consists of a square enclosure, with a circular goal area (green) in the center. A U-shaped obstacle (red) makes the task harder by forcing turns on trajectories from three out of the four possible starting locations (crosses). B: Color-coded trajectories of an example TD-LTP agent during the first 75 simulated trials. Early trials (blue) are spent exploring the maze and the obstacles, while later trials (green to red) exploit stereotypical behavior. C: Value map (color map) and policy (vector field) represented by the synaptic weights of the agent of panel B after 2000s simulated seconds. D: Goal reaching latency of agents using different learning rules. Latencies of 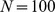 simulated agents per learning rule are binned by 5 trials (trials 1–5, trials 6–10, etc.). The solid lines shows the median of the latencies for each trial bin and the shaded area represents the 25th to 75th percentiles. For the R-max rule these all fall in the 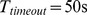 time limit after which a trial was interrupted if the goal was not reached. The R-max agent were simulated without a critic (see main text).

In the Morris water-maze, a rat or a mouse swims in an opaque-water pool, in search of a submerged platform. It is assumed that the animal is mildly inconvenienced by the water, and is actively seeking refuge on the platform, the reaching of which it experiences as a positive (rewarding) event. In our simulated navigation task, the learning agent (modeling the animal) is randomly placed at one out of four possible starting locations and moves in the two-dimensional space representing the pool (Figure 4A). Its goal is to reach the goal area (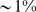 of the total area) which triggers the delivery of a reward signal and the end of the trial. Because the attractor dynamics in the pool of actor neurons make it natural for the agent to follow a straight line, we made the problem harder by surrounding the goal with a U-shaped obstacle so that from three out of four starting positions, the agent has to turn at least once to reach the target. Obstacles in the maze cause punishment (negative reward) when touched. Similar to what is customary in animal experiments, unsuccessful trials were interrupted (without reward delivery) when they exceeded a maximum duration 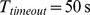.

During a trial, the synapses continually update their efficacies according to the learning rule, Eq. 17. When a trial ends, we simulate the animal being picked up from the pool by suppressing all place cell activity. This results in a quick fading away of all neural activity, causing the filtered Hebbian term in the learning rule to vanish and learning to effectively stop. After an inter-trial interval of 3s, the agent was positioned in a new random position, starting a new trial.

Figure 4B shows color-coded trajectories for a typical simulated agent. The naive agent spends most of the early trials (blue traces) learning to avoid walls and obstacles. The agent then encounters the goal, first at random through exploration, then repeatedly through reinforcement of the successful trajectories. Later trials (yellow to red traces) show that the agent mostly exploits stereotypical trajectories it has learned to reach the target.

We can get interesting insight into what was learned during the trials shown in Figure 4B by examining the weight of the synapses from the place cells to actor or critic neurons. Figure 4C shows the input strength to critic neurons as a color map for every possible position of the agent. This is in effect a “value map”: the value the agent attributes to each position in the maze. In the same graph, the synaptic weights to the actor neurons are illustrated by a vector field representing a “policy preference map”. It is only a preference map, not a real policy map because the input from the place cells (represented by the arrows) compete with the lateral dynamics of the actor network, which is history-dependent (not represented).

The value and policy maps that were learned are experience-dependent and unique to each agent: the agent shown in Figure 4B and C first discovered how to reach the target from the “north” (N) starting position. It then discovered how to get to the N position from starting positions E and W, and finally to get to W from S. It has not however discovered the way from S to E. For that reason the value it attributes to the SE quarter is lower than to the symmetrically equivalent quarter SW. Similarly the policy in the SE quarter is essentially undefined, whereas the policy in the SW quarter clearly points in the correct direction.

Figure 4D shows the distribution of latency – the time it takes to reach the goal – as a function of trials, for 100 agents. Trials of naive agents end after an average of 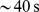 (trials were interrupted after 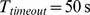). This value quickly decreases for agents using the TD-LTP learning rule (green), as they learn to reach the reward reliably in about 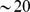 trials.

We previously remarked that the TD-LTP rule of Eq. 17 is similar to TD-STDP, the TD-modulated version of the R-STDP rule [6], [30], [31], at least in form. To see whether they are also similar in effect, in our context, we simulated agents using the TD-STDP learning rule (for both critic and actor synapses). The blue line in Figure 4D show that the performance was only slightly worse than that of the TD-LTP rule, confirming our finding on the linear track that both rules are functionally equivalent.

Policy gradient methods [5] follow a very different approach to reinforcement learning to TD methods. A policy gradient method for spiking neurons is R-max [4], [6], [32], [38], [39]. In short, R-max works by calculating the covariance between Hebbian pre-before-post activity and reward. Because this calculation relies on averaging those values over many trials, R-max is an inherently slow rule, typically learning on hundreds or thousands of trials. One would therefore expect that it can't match the speed of learning of TD-LTP or TD-STDP. Another difference of R-max with the other learning rules studied is that it does not need a critic [32]. Therefore we simulated an agent using R-max that only had an actor, and replaced the TD-signal by the reward, 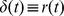. The red line of Figure 4 show that, as expected, R-max agents learn much slower than previously simulated agent, if at all: learning is actually so slow, consistent with the usual timescales for that learning rule, that it can't be seen in the graph because this would require much longer simulations.

One might object that using the R-max rule without a critic is unfair, and that it might benefit from a translation into a R-max rule with R = TD, by replacing the reward term by the 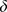 error, as we did for R-STDP. But this overlooks two points. Firstly, such a “TD-max” rule could not be used to learn the critic: by construction, it would tend to maximize the TD error, which is the opposite of what the critic has to achieve. Secondly, even if one were to use a different rule (e.g. TD-LTP) to learn the critic, this would not solve the slow timescale problem. We experimented with agents using a “TD-max” actor while keeping TD-LTP for the critic, but could not find notable improvement over agents with an R-max actor (data not shown).

### Acrobot Task

**Figure 5:**
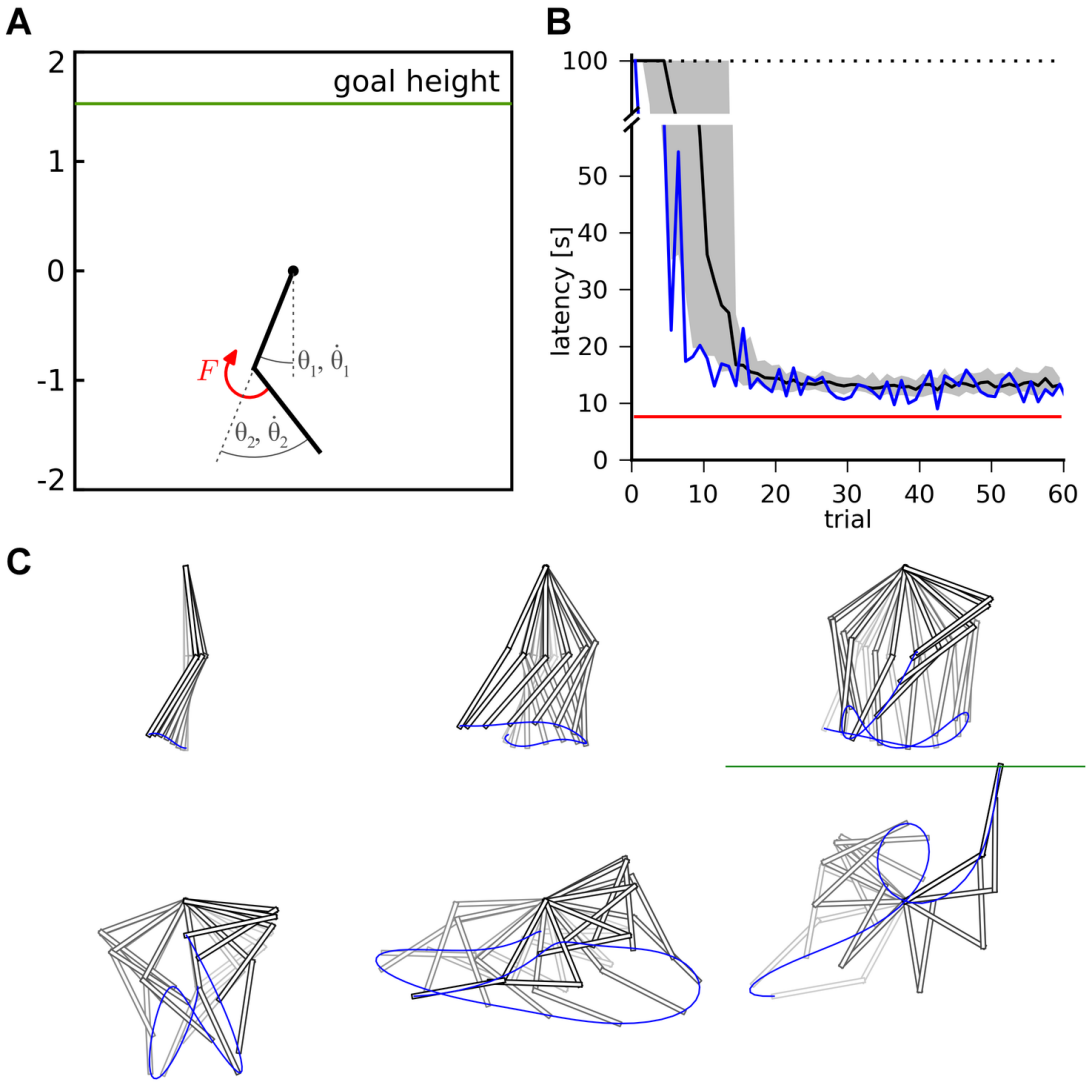
Acrobot task. A: The acrobot swing-up task figures a double pendulum, weakly actuated by a torque 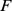 at the joint. The state of the pendulum is represented by the two angles 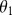 and 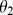 and the corresponding angular velocities 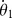 and 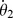. The goal is to lift the tip above a certain height 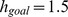 above the fixed axis of the pendulum, corresponding to the length 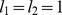 of the segments. B: Goal reaching latency of 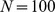 TD-LTP agents. The solid line shows the median of the latencies for each trial number and the shaded area represents the 25th to 75th percentiles of the agents performance. The red line represents a near-optimal strategy, obtained by the direct search method (see Models). The blue line show the trajectory of one of the best amongst the 100 agents. The dotted line shows the 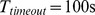 limit after which a trial was interrupted if the agent did not reach the goal. C: Example trajectory of an agent successfully reaching the goal height (green line).

Having shown that our actor-critic system could learn a navigation task, we now address a task that requires higher temporal accuracy and higher dimensional state spaces. We focus on the acrobot swing-up task, a standard control task in the reinforcement control literature. Here, the goal is to lift the outermost tip of a double pendulum under the influence of gravity above a certain level, using only a weak torque at the joint (Figure 5A). The problem is similar to that of a gymnast hanging below an horizontal bar: her hands rotate freely around the bar, and the only way to induce motion is by twist of her hips. While a strong athlete might be able to lift her legs above her head in a single motion, our acrobot is too weak to manage this. Instead, the successful strategy consists in moving the legs back and forth to start a swinging motion, building up energy, until the legs reach the sufficient height.

The position of the acrobot is fully described by two angles, 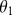 and 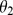 (see Figure 5A). However, the swinging motion required to solve the task means that even in the same angular position, different actions (torque) might be required, depending on whether the system is currently swinging to the left or to the right. For this reason, the angular velocities 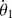 and 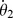 are also important variables. Together, these four variables represent the state of the agent, the four-dimensional equivalent of the x–y coordinates in the navigation task. Just as in the water-maze case, place cells firing rates were tuned to specific points in the 4-dimensional space.

Again similar to the maze navigation, the choice of the action (in this case the torque exerted on the pendulum joint) is encoded by the population vector of the actor neurons. The only two differences to the actor in the water-maze are that (i) the action is described by a single scalar and (ii) the action neuron attractor network is not on a closed ring anymore, but rather an open segment, encoding torques 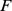 in the range 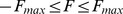.

Several factors make the acrobot task harder than the water-maze navigation task. First, the state space is larger, with four dimensions against two. Because the number of place cells we use to represent the state of the agent grows exponentially with the dimension of the state space, this is a critical point. A larger number of place cells means that each is visited less often by the agent, making learning slower. At even higher dimensions, at some point the place cells approach is expected to fail. However, we want to show that it can still succeed in four dimensions.

A second difficulty arises from the faster dynamics of the acrobot system with respect to the neural network dynamics. Although in simulations we are in full control of the timescales of both the navigation and acrobot dynamics, we wish to keep them in range with what might naturally occur for animals. As such the acrobot model requires fast control, with precision on the order of 100ms. Finally, the acrobot exhibits complex dynamics, chaotic in the control-less case. Whereas the optimal strategy for the navigation task consists in choosing an action (i.e., a direction) and sticking to it, solving the acrobot task requires precisely timed actions to successfully swing the pendulum out of its gravity well.

In spite of these difficulties, our actor-critic network using the TD-LTP learning rule is able to solve the acrobot task, as Figure 5B shows. We compared the performance to a near-optimal trajectory [40]: although our agents are typically twice as slow to reach the goal, they still learn reasonable solutions to the problem. Because the agents start with mildly random initial synaptic weights (see Models) and are subject to stochasticity, their history, and thus their performance, vary; the best agents have performance approaching that of the optimal controller (blue trace in Figure 5B).

### Cartpole Task

**Figure 6:**
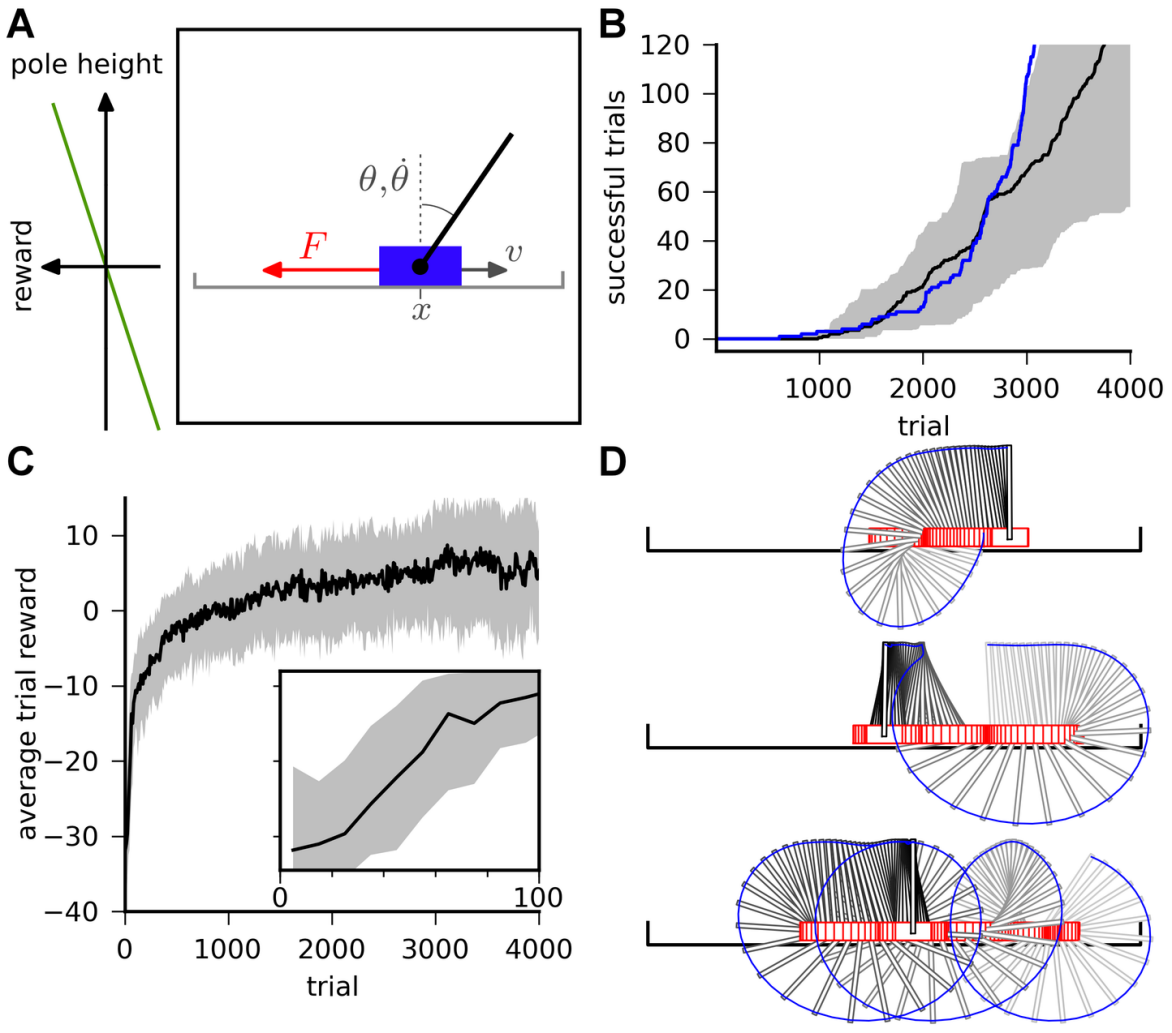
Cartpole task. A: Cartpole swing-up problem (schematic). The cart slides on a rail of length 5, while the pole of length 1 rotates around its axis, subject to gravity. The state of the system is characterized by 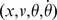, while the control variable is the force 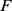 exerted on the cart. The agent receives a reward proportional to the height of the pole's tip. B: Cumulative number of “successful” trials as a function of total trials. A successful trial is defined as a trial where the pole angle was maintained up (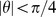) for more than 10s, out of a maximum trial length 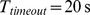. The black line shows the median, and the shaded area represents the quartiles of 20 TD-LTP agents' performance, pooled in bins of 10 trials. The blue line shows the number of successful trials for a single agent. C: Average reward in a given trial. The average reward rate 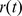 obtained during each trial is shown versus the trial number. After a rapid rise (inset, vertical axis same as main plot), the reward rises in a much slower timescale as the agents learn the finer control needed to keep the pole upright. The line and the area represent the median and the quartiles, as in B. D: Example agent behavior after 4000 trials. The three diagrams show three examples of the same agent recovering from unstable initial conditions (top: pole sideways, center: rightward speed near rail edge, bottom: small angle near rail edge).

We next try our spiking neuron actor-critic network on a harder control task, the cartpole swing-up problem [19]. This is a more difficult extension of cartpole balancing, a standard task in machine learning [18], [41]. Here, a pole is attached to a wheeled cart, itself free to move on a rail of limited length. The pole can swing freely around its axle (it doesn't collide with the rail). The goal is to swing the pole upright, and, ideally, to keep it in that position for as long as possible. The only control that can be exerted on the system is a force 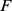 on the cart (Figure 6A). As in the acrobot task, four variables are needed to describe the system: the position 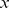 of the cart, its velocity 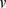, and the angle 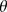 and angular velocity 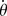 of the pole. We define a successful trial as a trial where the pole was kept upright (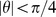) for more than 10 s, out of a maximum trial length of 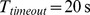. A trial is interrupted and the agent is punished for either hitting the edges of the rail (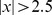) or “over-rotating” (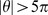). Agents are rewarded (or punished) with a reward rate 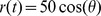.

The cartpole task is significantly harder than the acrobot task and the navigation task. In the two latter ones, the agent only has to reach a certain region of the state space (the platform in the maze, or a certain height for the acrobot) to be rewarded and to cause the end of the trial. In contrast, the agent controlling the cartpole system must reach the region of the state space corresponding to the pole being upright (an unstable manifold), and must learn to fight adverse dynamics to stay in that position.

For this reason learning to successfully control the cartpole system takes a large number of trials. In Figure 6B, we show the number of successful trials as a function of trial number. It takes the “median agent” (black line) on the order of 3500 trials to achieve 100 successful trials. This is slightly worse but on the same order of magnitude as the (non-neural) actor-critic of [19], which needs 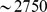 trials to reach that performance.

The evolution of average reward by trial (Figure 6C) shows that agents start with a phase of relatively quick progression (inset), corresponding to the agents learning to avoid the immediate hazard of running into the edges of the rail. This is followed by slower learning, as the agents learn to swing and control the pole better and better. To ease the long learning process we resorted to variable learning rates for both the actor and critic on the cartpole task: we used the average recent rewards obtained to choose the learning rate (see Models). More precisely, when the reward was low, agents used a large learning rate, but when performance improved, the agents were able to learn finer control strategies with a small learning rate. Eventually agents manage fine control and easily recover from unstable situations (Figure 6D). Detailed analysis of the simulation results showed that our learning agents suffered from noise in the actor part of the network, hampering the fine control needed to keep the pole upright. For example, the agent in Figure 6D has learned how to recover from a falling pole (top and middle plots) but will occasionally take more time than strictly necessary to bring the pole to a vertical standstill (bottom plot). The additional spike firing noise in our spiking neuron implementation could potentially explain the performance difference with the actor-critic in [19].

## Discussion

In this paper, we studied reward-modulated spike-timing-dependent learning rules, and the neural networks in which they can be used. We derived a spike-timing-dependent learning rule for an actor-critic network and showed that it can solve a water-maze type learning task, as well as acrobot and cartpole swing-up tasks that both require mastering a difficult control problem. The derived learning rule is of high biological plausibility and resembles the family of R-STDP rules previously studied.

### Biological Plausibility

**Figure 7:**
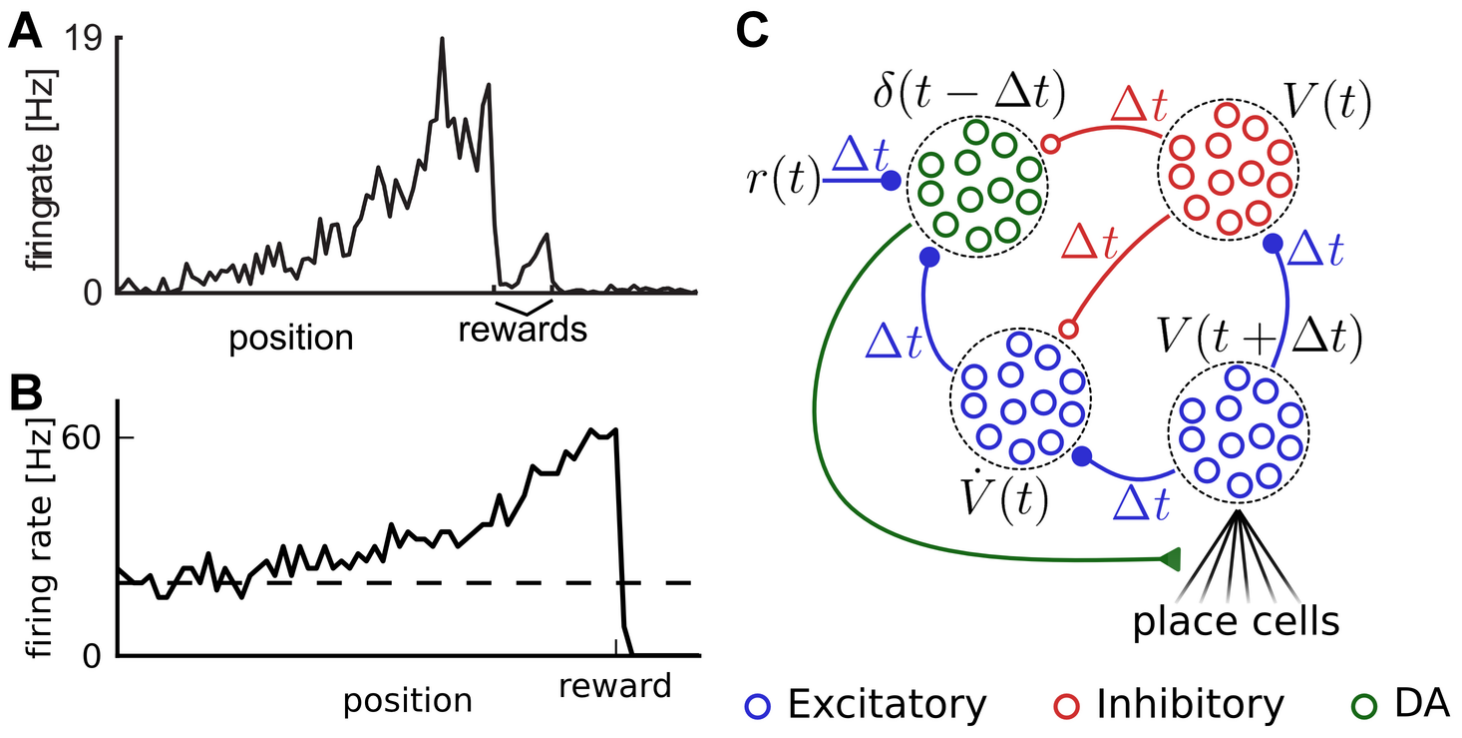
Biological plausibility. A: Firing rate of rat ventral striatum “ramp cells” during a maze navigation task. In the original experiment, the rat was rewarded in two different places, first by banana flavored food pellets, corresponding to the big drop in activity, then by neutral taste food pellets, corresponding to the end of small ramp. Adapted from van der Meer and Redish [44]. B: Firing rate of a single critic neuron in our model from the linear track task in Figure 2C. The dashed line indicates the firing rate 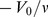 (Eq. 12) corresponding to 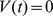. C: Putative network to calculate the TD error using synaptic delays. The lower right group of neurons corresponds to the critic neurons we considered in this paper. Each group of neurons gets its input delayed by the amount of the synaptic delay 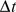. Provided the synapses have the adequate efficacies (not shown), this allows the calculation of 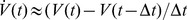 and the TD error 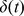.

Throughout this study we tried to keep a balance between model simplicity and biological plausibility. Our network model is meant to be as simple and general as possible for an actor-critic architecture. We don't want to map it to a particular brain structure, but candidate mappings have already been proposed [42], [43]. Although they do not describe particular brain areas, most components of our network resemble brain structures. Our place cells are very close to – and indeed inspired by – hippocampal place cells [22]. Here we assume that the information encoded in place cells is available to the rest of the brain. Actor neurons, tuned to a particular action and linked to the animal level action through population vector coding are similar to classical models of motor or pre-motor cortices [37]. So-called “ramp” neurons of the ventral striatum have long been regarded as plausible candidates for critic neurons: their ramp activity in the approach of rewards matches that of the theoretical critic. If one compares experimental data (for example Figure 7A, adapted from van der Meer and Redish [44]) and the activity of a typical critic neuron (Figure 7B), the resemblance is striking. The prime neuromodulatory candidate to transmit the global TD error signal to the synapses is dopamine: dopaminergic neurons have long been known to exhibit TD-like activity patterns [7], [45].

A problem of representing the TD error by dopamine concentration is that while the theoretically defined 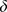 error signal can be positive as well as negative, dopamine concentration values [DA] are naturally bound to positive values [46]. This could be circumvented by positing a non-linear relation between the two values (e.g., 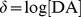) at the price of sensitivity changes over the 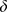 range. Even a simpler, piecewise linear scheme 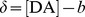 (where 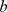 is the baseline dopamine concentration) would be sufficient, because learning works as long as the *sign* of the TD error is correct.

Another possibility would be for the TD error to be carried in the positive range by dopamine, and in the negative range by some other neuromodulator. Serotonin, which appears to play a role similar to negative TD errors in reversal learning [47], is a candidate. On the other hand this role of serotonin is seriously challenged by experimental recordings of the activity of dorsal raphe serotonin neurons during learning tasks [48], [49], which fail to show activity patterns corresponding to an inverse TD signal.

One of the aspects of our actor-critic model that was not implemented directly by spiking neurons but algorithmically, is the computation of the TD signal which depends on the reward, the value function and its derivative. In our model, this computation is crucial to the functioning of the whole. Addition and subtraction of the reward and the value function could be done through concurrent excitatory and inhibitory input onto a group of neurons. Similarly, the derivative of the value function could be done by direct excitation by a signal and delayed (for example by a an extra synapse) inhibition by the same signal (see example in Figure 7C). It remains to be seen whether such a circuit can effectively be used to compute a useful TD error. At any rate, connections from the the ventral striatum (putative critic) to the substantia nigra pars compacta (putative TD signal sender) show many excitatory and inhibitory pathways, in particular through the globus pallidus, which could have precisely this function [50].

### Limitations

A crucial limitation of our approach is that we rely on relatively low-dimensional state and action representations. Because both use similar tuning/place cells representations, the number of neurons to represent these spaces has to grow exponentially with the number of dimensions, an example of the curse of dimensionality. While we show that we can still successfully solve problems with four-dimensional state description, this approach is bound to fail sooner or later, as dimensionality increases. Instead, the solution probably lies in “smart” pre-processing of the state space, to delineate useful and reward-relevant low dimensional manifolds on which place cells could be tuned. Indeed, the representation by place cells can be learned from visual input with thousands of “retinal” pixels, using standard unsupervised Hebbian learning rules [20], [51], [52].

Moreover, TD-LTP is derived with the assumption of sparse neural coding, with neurons having narrow tuning curves. This is in contrast to covariance-based learning rules [53], such as R-max [4], [6], [38], [39] which can, in theory, work with any coding scheme, albeit at the price of learning orders of magnitude slower.

### Synaptic Plasticity and Biological Relevance of the Learning Rule

Although a number of experimental studies exist [11]–[14], [54] targeting the relation between STDP and dopamine neuromodulation, one is at pain to draw precise conclusions as to how these two mechanism interplay in the brain. As such, it is hard to extract a precise learning rule from the experimental data. On the other hand, we can examine our TD-LTP learning rule in the light of experimental findings and see whether they match, i.e., whether a biological synapse implementing TD-LTP would produce the observed results.

Experiments combining various forms of dopamine or dopamine receptor manipulation with high-frequency stimulation protocols at the cortico-striatal synapses provide evidence of an interaction between dopamine and synaptic plasticity [8]–[11]. While these experiments are too coarse to resolve the spike-timing dependence, they form a picture of the dopamine dependence: it appears that at high concentration the effect of dopamine paired with high-frequency stimulation is the induction of long-term potentiation (LTP), while at lower concentrations, long-term depression (LTD) is observed. At a middle “baseline” concentration, no change is observed. This picture is consistent with TD-LTP or TD-STDP if one assumes a relation 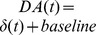 between the dopamine concentration 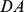 and the TD error.

The major difference between TD-LTP and TD-STDP is the behavior of the rule on post-before-pre spike pairings. While TD-LTP ignores these, TD-STDP causes LTD (resp. LTP) for positive (resp. negative) neuromodulation. Importantly this doesn't seem to play a large role for the learning capability of the rule, i.e., the pre-before-post is the only crucial part. This is interesting in the light of the study by Zhang et al. [13] on hippocampal synapses, that finds that extracellular dopamine puffs reverse the post-before-pre side of the learning window, while strengthening the pre-before-post side. This is compatible with the fact that polarity of the post-before-pre side of the learning window is not crucial to reward-based learning, and might serve another function.

One result predicted by both TD-LTP and TD-STDP and that has not, to our knowledge, been observed experimentally, is the sign reversal of the pre-before-post under negative reward-prediction-error signals. This could be a result of the experimental challenges required to lower dopamine concentrations without reaching pathological levels of dopamine depression. However high-frequency stimulation-based experiments show that a reversal of the global polarity of long-term plasticity indeed happens [8], [11]. Moreover, a study by Seol et al. [54] of STDP induction protocols under different (unfortunately not dopaminergic) neuromodulators shows that both sides of the STDP learning window can be altered in both polarity and strength. This shows that a sign change of the effect of the pre-then-post spike-pairings is at least within reach of the synaptic molecular machinery.

Another prediction that stems from the present work is the existence of eligibility traces, closing the temporal gap between the fast time requirements of STDP and delayed rewards. The concept of eligibility traces is well explored in reinforcement learning [1], [5], [55], [56], and has previously been proposed for reward-modulated STDP rules [6], [30]. Although our derivation of TD-LTP reaches an eligibility trace by a different path (filtering of the spike train signal, rather than explicitly solving the temporal credit assignment problem), the result is functionally the same. In particular, the time scales of the eligibility traces we propose, on the order of hundreds of milliseconds, are of the same magnitude as those proposed in models of reward-modulated STDP [6], [30]. Direct experimental evidence of eligibility traces still lacks, but they are reminiscent of the synaptic tagging mechanism [57]. Mathematical models of tagging [58], using molecular cascades with varying timescales, provide an example of how eligibility traces could be implemented physiologically.

### Insights for Reward-Modulated Learning in the Brain

One interesting result of our study, is the fact that although our TD signal properly “teaches” the critic neurons the value function and back-propagates the reward information to more distant points, it is difficult to see the back-propagation in the time course of the TD signal itself. The reason for this is that the signal is drowned in rapid fluctuations. If one were to record a single neuron representing the TD error, it would probably be impossible to reconstruct the noiseless signal, except with an extremely high number of repetitions under the same conditions. This might be an explanation for the fact that the studies by Schultz and colleagues (e.g., [45]) repeatedly fail to show back-propagation of the TD error, even though dopamine neurons seem to encode such a signal.

In this study, TD-STDP (and TD-LTP) is used in a “gated-Hebbian” way: a synapse between A and B should be potentiated if it witnessed pre-before-post pairings and the TD signal following later is positive. This is fundamentally different from the role of the reward-modulated version of that learning rule (R-STDP) in [32], where it is used to do covariance-based learning: a synapse between A and B should be potentiated if it witnesses positive correlation between pre-before-post pairings and a success signal, *on average*. One consequence of this is the timescale of learning: while TD-based learning takes tens of trials, covariance based learning typically requires hundreds or thousands of trials. The other side of the coin is that covariance-based learning is independent of the neural coding scheme, while TD-based learning requires neural tuning curves to map the relevant features prior to learning. The fact that the mathematical structure of the learning rule (i.e., a three-factor rule where the third factor “modulates” the effect of pre-post coincidences [59]) is the same in both cases is remarkable, and one can see the advantage that the brain might have had to evolve such a multifunctional tool — a sort of “Swiss army knife” of synaptic plasticity.

## Models

### Neuron Model

For the actor and critic neurons we simulated a simplified spike response model (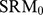, [60]). This model is a stochastic variant of the leaky integrate-and-fire neuron, with the membrane potential of neuron of 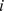 given by
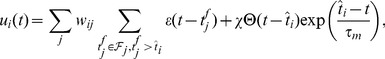
(18)where 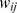 is the efficacy of the synapse from neuron 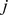 to neuron 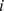, 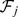 is the set of firing times of neuron 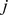, 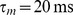 is the membrane time constant, 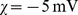 scales the refractory effect, 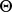 is the Heaviside step function and 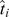 is the last spike of neuron 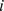 prior to 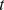. The EPSP is described by the time course
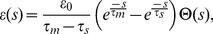
(19)where 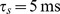 is the synaptic rise time and 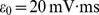 is a scaling constant, and 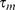 is the membrane time constant, as in Eq. 18. Given the membrane potential 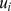, spike firing in the 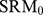 is an inhomogeneous Poisson process: at every moment the neuron has a probability of emitting a spike, according to an instantaneous firing rate
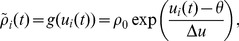
(20)where 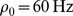, 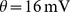 and 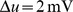 are constants consistent with experimental values [61]. In the limit 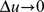, the 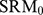 becomes a deterministic leaky integrate-and-fire neuron.

### Navigation Task

The Morris water-maze pool is modeled by a two-dimensional plane delimited by a square wall. The position 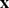 of the agent on the plane obeys
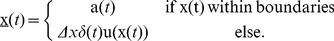
(21)When the agent is within boundaries it moves with speed 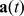, as defined by the actor neurons' activity (Eq.29). Whenever the agent encounters a wall, it instantly “bounces” back a distance 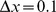 along unitary vector 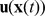, which points inward, perpendicular to the obstacle surface. Every “bumping” against a wall is accompanied by a punishing, negative reward 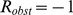 delivery (see reward delivery dynamics below).

We used two variants of the navigation task. The linear track is a narrow rectangle of size 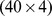 centered around the origin, featuring a single starting position in 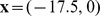 and a wide goal area (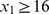) on the opposite side. Because the goal of this setup is to study critic learning, the action is clamped to a fixed value 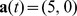, so that the agent runs toward the goal at a fixed speed.

The second variant is the navigation maze with obstacle. It consists of a square area of size 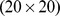 centered around the origin, with four starting positions at 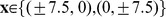. The goal area is a circle of radius 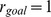 centered in the middle of the maze. The goal is surrounded on three sides by a U-shaped obstacle (width of each segment: 2, length: 10).

In both variants, place cells centers 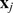 are disposed on a grid (blue dots on Figure 1), with spacing 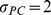 coinciding with the width of the place fields. The outermost centers lie a distance 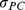 outside the maze boundaries. This ensures a smooth coverage of the whole state space. In the case of the maze, the place cell grid consists of 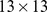 centers. For the linear track setup, the grid has 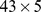 centers.

Trials start with the agent's position 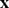 being randomly chosen from one out of four possible starting positions. The place cells, indexed by 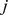, are inhomogeneous Poisson processes. After a trial starts, the place cells' instantaneous firing rates are updated to
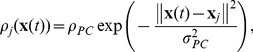
(22)where 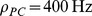 is a constant regulating the activity of the place cells, 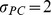 is the place cells separation distance and the 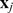 are the place cells centers. The presynaptic activity in the place cells generates activity in the post-synaptic neurons of the critic and the actor with a small delay caused by the rise time of EPSPs.

The value function 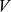 is calculated according to Eqs 12 and 13, with parameters 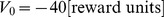 and 
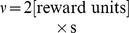
. Because 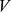 is delayed by the rise time 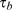 of the 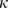 kernel, at the start of a trial the TD error 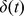 is subject to large, boundary effect transients. To cancel these artifacts, we clamp the TD error to 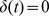, for the first 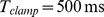 of each trial. We use a reward discount time constant 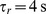.

The goal of the agent is to reach the circular area which represents the submerged platform of the water-maze. When the agent reaches this platform, a positive reward 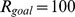 is delivered, the trial ends and the agent is put in a so-called “neutral state”, which models the removal of the animal from the experiment area. The effects of this is (i) the place cells corresponding to the maze become silent, presumably replaced by other (not modeled) place cells, and (ii) the expectation of the animal becomes neutral, and therefore its value function goes to zero. So at the end of a trial, we turn off place cell activity (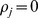), and the value function is no longer given by Eq. 13, but decays exponentially to 0 with time constant 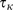 from its value at the time of the end of the trial. Importantly, synaptic plasticity continues after the end of the trial, so that the effect of 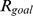 affects the synaptic weight even though its delivery takes place in the neutral state. Additionally, a trial can end without the platform being reached: if a trial exceeds the time limit 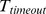, it is declared a failed trial, and interrupted with the agent put in the neutral state, just as in the successful case, but without reward being delivered.

According to Eq. 3, rewards are given to the agent as a reward rate. This reflects the fact that “natural” rewards, and reward consumption, are spread over time, rather than point-like events. So we translate absolute rewards (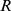) to a reward rate (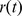), calculated as the difference of two decaying “traces” obeying dynamics
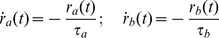
(23)i.e.,
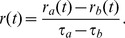
(24)At most times, the reward is close to 0. Reward is delivered only when some event (goal reached or collision against an obstacle) occurs. The delivery of a reward 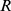 happens through instantaneous update of the traces
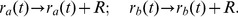
(25)The resulting effect is a subsequent positive excursion of 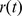, with rise time 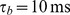 and fall time 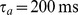, which, integrated over time, amounts to 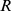.

### Acrobot Task

In the acrobot task, the position of the pendulum is described by two angles: 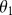 is the angle between the first segment of the pendulum and the vertical, and 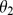 is the angle between the second segment and an imaginary prolongation of the first (Figure 5A). When 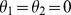, the pendulum hangs down. Critical to solving the task are also the angular velocities 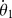 and 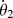. As in the maze navigation case, place cells tuned to specific centers are used to represent the state of the acrobot. We transform the angular velocities 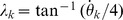, 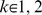. This allows a fine resolution over small velocities, while maintaining a representation of higher velocities with a small number of place cells. The state 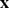 is represented by the four variables 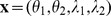.

The place cells centers are disposed on a 4-dimensional grid defined by indexes 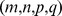, such that 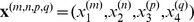 with
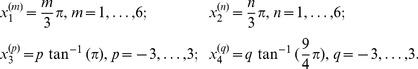
(26)This yields a total of 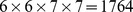 centers. The activity of a place cell with center 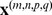 is defined by
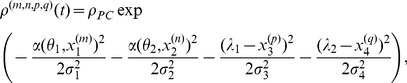
(27)where 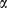 is a function returning the difference between two angles modulo 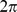 in the range 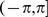 and the place cell widths 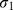 to 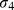 correspond to the grid spacing as in Eq. 26.

The acrobot dynamics obeys the following equations [1]:
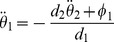


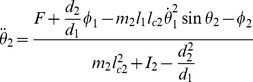


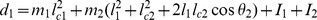


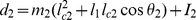


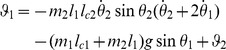


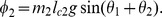
Here, 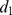, 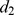, 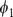 and 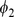 are convenience variables, 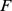 is the torque applied to the joint, 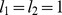 are the lengths of the segments, of mass 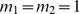, with moments of inertia 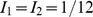 and lengths to the centers of mass 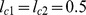, under the influence of gravity 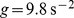. All dimensions except time are unit-less.

The goal is for the tip of the acrobot to reach a height 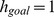 over the axis, i.e., fulfill the condition 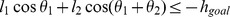. Once this happens, or the maximum trial time 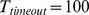 is reached, the trial ends. To entice the acrobot to do something, we give an ongoing punishment 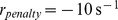 to the agent for not reaching the reward, to be compared with the reward 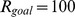 received at the goal. As in the water-maze case, we use a reward discount time constant 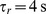.

Due to the larger number of place cells, we use less critic and actor neurons than in the maze case, respectively 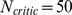 and 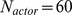, to reduce the number of synapses and the computational load.

To compare the performance of our agent against an “optimal” strategy, we use the direct search method [40]. The main idea behind the method is to search for the sequence of action that will maximize the system's total energy, with knowledge of the acrobot dynamics. To make the search computationally tractable, a few simplifications are made: actions are limited to the alternative 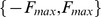, actions are only taken in steps of 100 ms, only a window of the next 10 steps is considered at a time, and the number of action switch in each window is limited to 2. Thus only 55 action sequences have to be examined, and the sequence that maximizes the total energy reached over the window, or reaches the goal height the sooner, is selected. The first action in that sequence is chosen as the action for the next step and the whole procedure is repeated with the window shifted by one step. The goal height reaching latency found with this method was 7.66s (red line in Figure 5B).

### Cartpole Task

The position of the cartpole system is described by the cart position 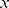, the cart velocity 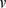, the angle of the pole with the vertical 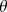 (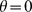 corresponds to the pole pointing upwards) and the angular velocity 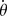; these form the state vector 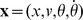. Similar to the acrobot, the place cells for the cartpole problem are regularly disposed on a four-dimensional grid of 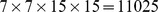 cells. The location of a place cell with index 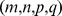 is at location 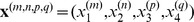 with
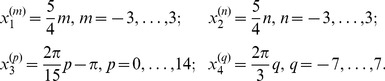
(28)The activity of a place cell is defined in a way analog to Eq. 27. The variance of the gaussian place fields is diagonal 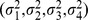, where 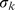 corresponds to the grid spacing in dimension 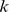.

The dynamics of the cartpole are [62]:
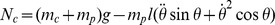


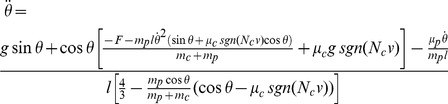


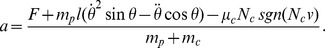
Here *a = v* is the acceleration of the cart, 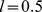 is half the pole's length, 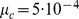 and 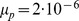 are coefficients of friction of the cart on the track and of pole rotation respectively. The cart, with mass 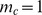, and the pole, with mass 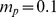, are subject to the acceleration of gravity 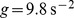. As in the acrobot case, all dimensions except time are unit-less.

Following [19], the agent is rewarded continuously depending on the current height of the pole with 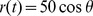, and the reward discount time constant is set to 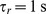. If the cart runs off its rail (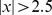) or over-rotates (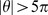) the trial is ended and a negative reward 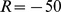 is given. A trial ends without reward after 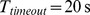. When a new trial starts, the position of the system is initialized with a random 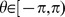 and 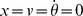.

### Actor Dynamics

In population vector coding, each actor neuron 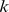 “votes” for its preferred action 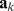 in the action space, by firing an action potential. An action vector is obtained by averaging the product of the instantaneous firing rate 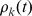 (see Eq. 12) and the action vector of each neuron, i.e.
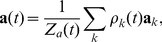
(29)where 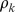 is defined as
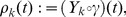
(30)with filter
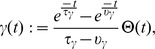
with 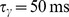 and 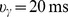 being filtering time constants. The term 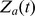 in Eq. 29 is a normalization term. In the case of the navigation task (two-dimensional action), it is equal to the number of actor neurons, 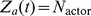. In the cases of the acrobot and the cartpole task (scalar action), 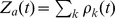.

We enforce a N-winner-takes-all mechanism on the action neurons by imposing “lateral” connectivity between the action neurons: action neurons coding for similar actions excite each other, while they inhibit the neurons coding for dissimilar actions. The synaptic weight between two action neurons 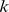 and 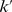 is
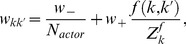
(32)where 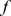 is a lateral connectivity function. This is zero for 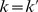, peaks for 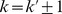 and monotonously decreases towards 0 as 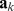 and 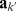 diverge. 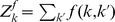 is a normalization constant. The parameters 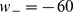 and 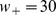 regulating the recurrent connections were manually tuned: the lateral connectivity has to be strong enough so that there is always exactly one “bump” of similarly tuned neurons active whenever the action neurons receive some excitation from the place cells, but not so strong that it completely dominates the feed-forward input from the place cells.

The preferred vectors 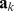 of the action neurons and the function 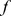 are dependent on the learning task. In the case of the maze navigation task, the preferred action vectors are 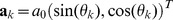 where 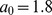 is a constant representing the agent velocity per rate unit and 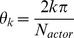, for 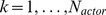. The 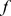 function was chosen as
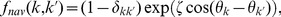
(33)with 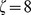.

In the case of the acrobot and cartpole tasks, the action vectors are 
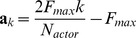
. For the acrobot 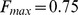 represents the maximum torque that the agent can exert and for the cartpole task 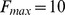 is the maximum force on the cart. The lateral connectivity function 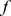 in both cases was chosen as
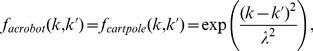
(34)with 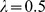. Additionally, we algorithmically constrain the torque exerted by the agent to the domain 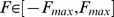. This models the limited strength of the agent's “muscles”.

### Other Reward-Modulated Synaptic Learning Rules

In R-STDP [6], [30]–[32], the effects of classic STDP are modulated by a neuromodulatory signal 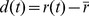, where 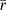 is a constant baseline. We transformed the reward-modulated R-STDP into the TD-modulated rule TD-STDP by replacing the 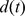 with 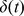. The TD-STDP rule can be written as
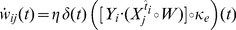
(35)where the STDP learning window is
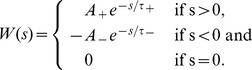
The eligibility trace kernel 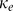 is the result of an exponential decay, i.e., 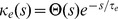, with time constant 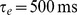. The positive constants 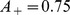 and 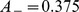 govern the size of the pre-before-post and post-before-pre parts of the learning window respectively, and the time constants 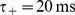 and 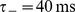 determine their timing requirement.

R-max [4], [6], [32], [38] is a reward-modulated learning rule derived from policy gradient principles [5]. It can be written as
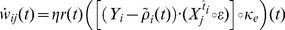
(36)where 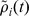 is the instantaneous firing rate of neuron 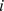, as defined in Eq. 20.

### Simulation Details

Initial values of the synaptic weights to both critic and actor were randomly drawn from a normal distribution with mean 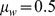 and standard deviation 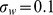. These values ensured an initial value function 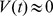 and reasonable action neuron activity prior to learning.

For all learning rules, synaptic weights were algorithmically constrained to the range 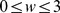, to avoid negative or runaway weights. Learning rate values were manually adjusted (one value for actor and another one for critic synapses) to the value that yielded the best performance (as measured by the number of trials completed in 2.000s of simulated time). These values for the navigation and acrobot tasks are printed in Table 1. For the cartpole task, somewhat faster learning was achieved by using a variable learning rate
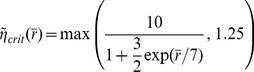
(37)for the critic, where 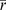 is a running average of past reward rates 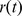, computed by filtering 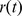 with an exponential window with time constant 50s. The actor learning rate was 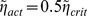.

**Table 1:** Learning rates.

Figure	Rule	Synapses	Value	Units
Figure 2C and D	TD-LTP	critic	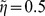	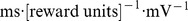
Figure 4B, C and D	TD-LTP	critic	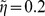	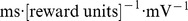
Figure 4B, C and D	TD-LTP	actor	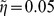	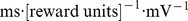
Figure 4D	TD-STDP	critic	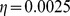	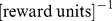
Figure 4D	TD-STDP	actor	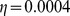	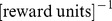
Figure 4D	R-max	actor	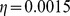	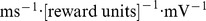
Figure 5B and C	TD-LTP	critic	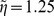	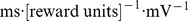
Figure 5B and C	TD-LTP	actor	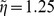	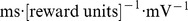

Numerical values of the learning rates for the different learning rules used in simulations.

All simulations were ran using Euler's method with time-step 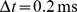, except for the acrobot and cartpole dynamics, simulated using 4th order Runge-Kutta with 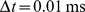.

### Derivation of 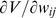


In this section we calculate the term 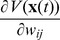, needed to derive Eq. 17. Using Eqs 12–13, and focusing on the synaptic weight 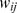 from 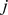 to 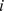, we find
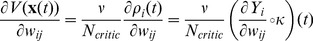
(38)where we used the fact that ρ*_i_*′**(** *t* **)** is independent of 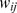 for 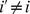. The derivative of the spike train 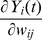 is ill-defined: in our stochastic neuron model, the spike train itself is independent of the synaptic weights. It is only the probability of the spike train actually being emitted by the neuron that depends on the weights. Therefore we replace 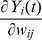 with 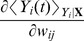, the expected value of the spike train 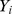 conditional on the input 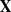. This yields
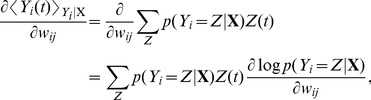
(39)where the sum is over all possible spike trains 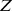 and 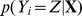 is the probability density of the spike train 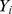 being equal to 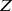. The probability density of that spike train 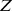, lasting from 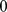 to 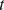, being produced by an SRM_0_ neuron receiving inputs 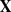 is [38]

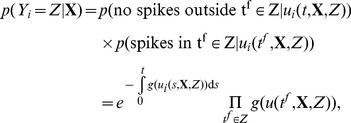
(40)where 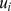 is the membrane potential (Eq. 18) and we have used Eq. 20. Combining Eqs 39 and 40 yields
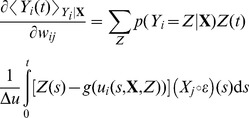
(41)The integration reflects the fact that the probability of a spike being emitted by the neuron at time 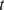 is dependent not only on recent presynaptic spikes, but also on the time of the last spike of neuron 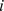, which in turn depends on its whole history.

It is not clear that, in our context, this history dependence is a desirable outcome. Two devices already take the spike train history into account. Firstly, the definition of the value function 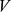 in the TD framework is conditional only on the current state, and not the long-term history. (This stems from the Markov decision process at the root of TD.) Secondly, the filtering of the spike train by 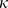 already ensures that the short-term history is remembered, making the integral over the history redundant.

For these reasons, we choose to neglect the neuron's history, and to perform the following substitution
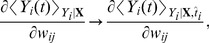
(42)i.e., we take the last spike time 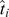 of neuron 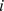 as given, and we ask how does the mean spiking at time 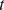 vary as a function of the synaptic weight 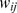. Therefore we have
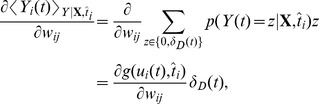
(43)where we have used the definition of the neuron's firing rate, Eq. 20, and 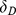 is the Dirac distribution. Using Eqs 18 yields
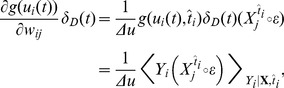
(44)where 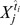 is the spike train of neuron 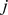 culled to times posterior to the spikes of neuron 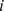, i.e., 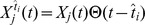, with 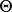 denoting the Heaviside step function. Wrapping up the steps from Eqs 38 and 42–44, we finally have
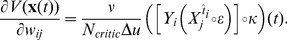
(45)


### Derivation of the Squared TD Gradient Learning Rule

**Figure 8:**
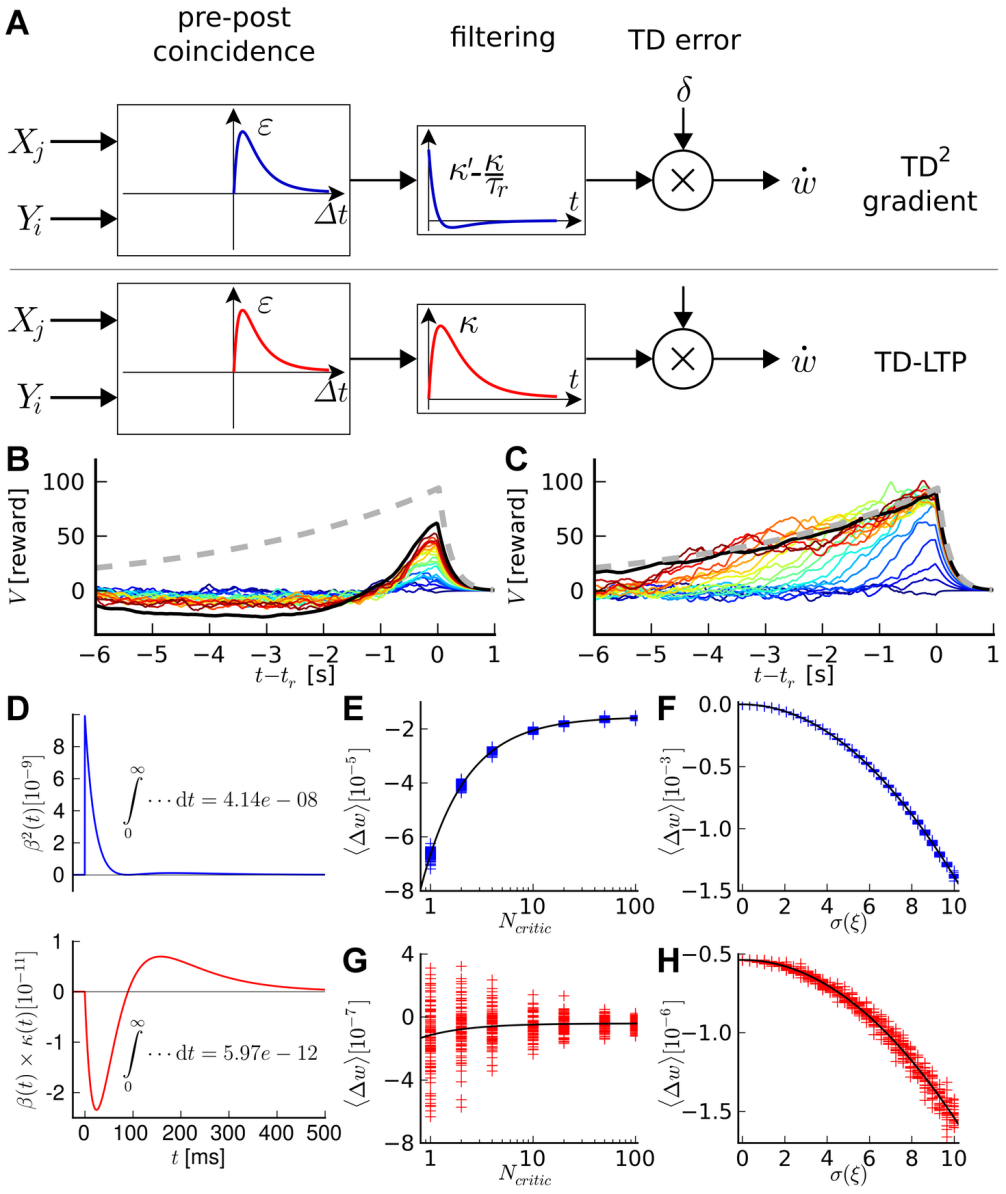
Alternative learning rule and nuisance term. A: Schematic comparison of the squared TD gradient learning rule of Eq. 46 and TD-LTP, similar to Figure 2A. B: Linear track task using the squared TD gradient rule. Same conventions as in Figure 2C. C: linear track task using the TD-LTP rule (reprint of Figure 2C for comparison). D: Integrands of the disturbance term for Poisson spike train statistics. Top: squared TD gradient rule. Bottom: TD-LTP rule. In each plot the numerical value under the curve is given. This corresponds to the contribution of each presynaptic spike to the nuisance term. E: Disturbance term dependence on 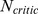 for the squared TD gradient rule. The mean weight change under initial conditions on an unrewarded linear track task with frozen weights, using the squared TD gradient learning rule, is plotted versus 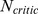, the number of neurons composing the critic. Each cross corresponds to the mean over a 200s simulation, the plot shows 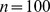 crosses for each condition. The line shows a fit of the data with 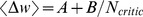, the dependence form suggested by Eq. 50. F: Same as E, for critic neurons using the TD-LTP learning rule. G, H: Same experiment as E and F, but using a rate neuron model with Gaussian noise of mean 0 and variance 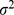. The line shows a fit with 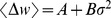, the dependence form suggested by Eq. 50.

In the Results section we derive a learning rule starting from Eq. 10. We also suggest that starting from a gradient descent on the squared TD error (Eq.17) should yield a valid learning rule. Here we derive such a learning rule. Combining Eq. 10, the definition of the TD error (Eq. 5) and the result of the previous section (Eq. 45), we find
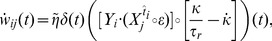
(46)where 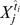 is the spike train of presynaptic neuron 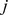. This learning rule has the same general form as the TD-LTP rule (Eq. 17): a “Hebbian” pre-before-post coincidence term is first temporally filtered, and then multiplied by the TD error with a term (Figure 8A). The difference lies in the extra 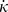 in the filter, which comes from a 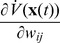 term. As Figure 8 suggests, the 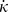 term largely dominates over 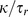. This is the consequence of our choice of a long discount time constant (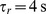) with a short (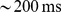) 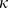 kernel.

### Noise Correlation Problem

Here we show, both analytically and in simulations, that the squared TD gradient learning rule of Eq. 46 suffers from a noise bias problem. This arises from the noise in the individual neurons estimating the value function, and is serious enough to prevent learning. To see this, we start by decomposing the spike train 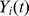 of a neuron into a mean and a noise term, i.e.
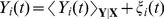
(47)where we have defined 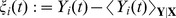, with the brackets 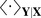 denoting expectation, i.e., averaging over all possible outcomes of critic neurons activity 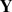 conditioned on the presynaptic neural activity 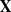. With this definition, we can rewrite Eq. 46 as
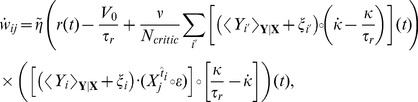
(48)where the 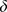 error has been spelled out explicitly (Eqs 5, 13 and 12). Eq. 48 suggests that quadratic terms 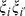 in the noise might play a role in the learning rule. Indeed, distributivity and use of the facts 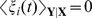 and 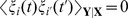 for 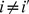 gives
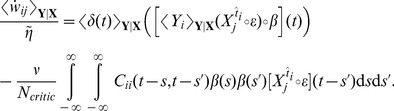
(49)Here we have defined the autocorrelation of the noise terms 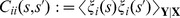, as well as 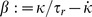 for brevity.

The first term in the right-hand side of Eq. 49 is analog to Eq. 46, with 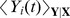 replacing 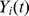, and 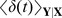 replacing 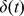. In effect this is a “mean” version of the learning rule: this is what one would get by replacing the stochastic spiking neurons in the model by analog, noiseless units with a similar exponential activation function.

The second term arises from the correlation of neuron noise in the TD term 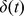 and the Hebbian component of the learning rule. This term is a function of the autocorrelation function of the postsynaptic neuron. This carries only indirect information about the postsynaptic firing (and thus the current value function 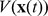) and no information about the reward 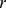. For this reason, we conjecture that this second element is a potentially problematic term, which we refer to as the “nuisance” term. This hypothesis is confirmed by linear track simulations using the learning rule Eq. 46, shown in Figure 8B. These indicate that the learning rule is unable to learn the task, contrary to TD-LTP (Figure 8C, same as Figure 2B). More precisely, the value functions learned by the squared TD gradient rule suffer from a negative “drag” term.

We next try to identify this negative “drag” with the nuisance term. Although there's no closed form expression for 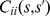, one can use the statistics of a Poisson process as a first order approximation. In that case 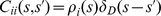 (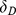 is the Dirac distribution) and Eq. 49 becomes
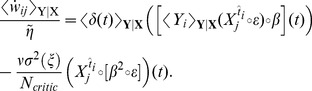
(50)


The last term on the right-hand side of Eq. 50 implies that, on average, each presynaptic spike in neuron 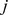 causes the synaptic weight 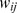 to depress by a fixed amount. This quantity increases with the variance 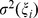 of the noise process, in this case the inhomogeneous Poisson process that drives the 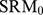 neuron, and inversely to the number 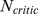 of critic neurons. The time course of the presynaptic spike effect is ruled by 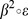, which is plotted in the top panel of Figure 8D. The aggregate nuisance effect on 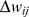 of a single presynaptic spike is proportional to the integral of 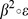 over time.

In Figure 8E, we explore the dependence of the nuisance term on 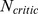 in numerical simulations. Eq. 50 suggests that the mean learning rule term should obey a relationship of the form
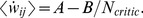
(51)Here 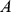 is the result of the “useful” part of the learning rule, and 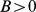 contains all the other dependencies of the nuisance term. We tested the 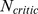 dependency by simulating agents with variable numbers of critic neurons in a linear track scenario. The setup was similar to that of Figure 2, except that the weights were frozen, i.e., we collected the value of the learning rule at each time step, but we didn't actually update the weights. The mean learning rule outcome for 200s of simulations are plotted in Figure 8E as crosses, against the number of critic neurons. The black line shows a fit of the data by Eq. 51: both are in good agreement.

From Eq. 50, we see that the nuisance term also depends on the variance 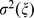 of the noise process. It is difficult to control the variance of our spiking neurons' noise process without also altering their firing rate and thus the result of the learning rule. To circumvent this difficulty, we turned to a rate model, where the single critic neuron's firing rate was
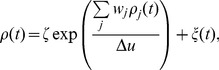
(52)where 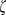 is a constant, the place cells rates 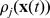 are defined in Eq. 22 and 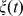 is a white noise process. Similar to the steps above, a gradient descent on 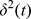 yields a learning rule of the form
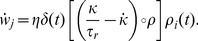
(53)Due to the noise component in 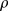, the learning rule suffers from the same noise-driven nuisance as the spiking version. This depends on the noise's variance 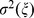, so that the mean weight change obeys
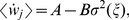
(54)where 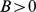. In Figure 8F, we use the rate-based model and rule in the same “frozen weights” linear track scenario as in Figure 8E. This time we looked at how the mean weight change varied as a function of the noise variance. Again, the data is well matched by a fit with Eq. 54 (black line), suggesting that the nuisance term behaves as expected.

### Noise Correlation in the TD-LTP Rule

In the preceding section we found that a noise correlation nuisance in the squared TD gradient learning rule causes it to be ineffective. However, the same actually should apply to the TD-LTP rule. Indeed, if we repeat the steps above leading to Eq. 50 for the learning rule TD-LTP, we get
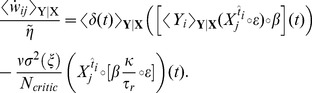
(55)


The only difference is the time course of the nuisance term, which is 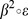 for the squared TD gradient rule versus 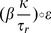 for TD-LTP. Figure 8D shows a plot of both expressions: because the TD-LTP expression is much smaller, these are plotted on different axes. As noted before, the integral of the nuisance is proportional to these time courses (shown on Figure 8D). The term for TD-LTP is more than three orders of magnitude smaller than that of the square TD gradient rule.

In Figure 8G and H, we repeat the experiments of Figure 8E and F, respectively. These show that the TD-LTP learning rule also suffers from a nuisance term, but that it is orders of magnitude smaller than for the squared TD gradient rule. As shown by Figure 8C and in the Results section, this nuisance is not sufficient to prevent TD-LTP from properly learning the value function 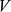.

### The Trouble with Continuous Q-Learning

In the Results section, we claim that 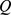-values based algorithms, such as Sarsa [24] and Q-Learning [23] are difficult to extend to continuous time in a neural network setting. Here we develop this argument.

In the discrete Sarsa algorithm, the agent maintains an estimation of the state-action 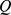-values. For an agent following the policy 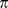, starting at time step 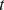 in state 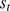 and executing action 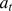, this is defined as the discounted sum over future rewards 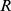:
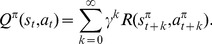
(56)Here 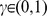 is a discount factor, and 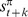 and 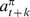 represent the future states and actions visited by the agent under policy 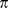. To learn 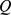-values approximations 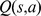 to the real 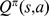, Sarsa suggests the following update rule at time step 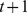:
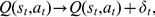
(57)where the TD error 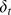 is defined as
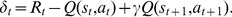
(58)


If one were to propose a continuous time version of Sarsa, one would start by redefining the state-action value function 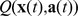 to continuous time t, similar to the value function of Eq. 3

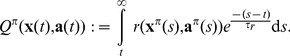
(59)Here 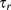 now plays the role of the discount factor 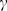. As we did for Eq. 5, we define the TD error on the 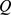-value by taking the derivative of Eq. 59

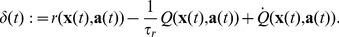
(60)To calculate the TD error, one therefore needs to combine the three terms in Eq. 60. We assume the reward 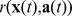 is given by the environment. Typically [16], [20], neural networks implementations of 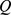-values based reinforcement learning consist of a number “action cells” neurons 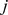, each tuned to a specific action 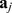 and rate-coding for the state-action values
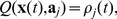
(61)where 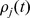 is neuron 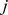
's firing rate. In that case, reading out the value 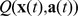 is thus simply a matter of reading the activity of the neuron 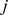 coding for the action 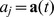 selected at time 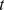.

Reading out the temporal derivative 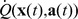 is harder to do in that context, because the currently chosen action is evolving all the time. For small 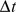, we can approximate
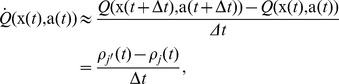
(62)where we also used Eq. 61 and identified the action neuron 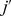 tuned to action 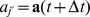.

The difficulty that arises in evaluating Eq. 62 is the following. It requires a system that can keep track of the two recent actions 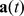 and 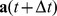, identify the relevant neurons 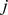 and 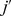, and calculate a difference of their firing rates. This is hard to envision in a biologically plausible setting. The use of an actor-critic architecture solves this problem by having a single population coding for the state-based value 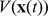 at all times.
